# The Effect of Tranexamic Acid on Neurosurgical Intervention in Spontaneous Intracerebral Hematoma: Data From 121 Surgically Treated Participants From the Tranexamic Acid in IntraCerebral Hemorrhage-2 Randomized Controlled Trial

**DOI:** 10.1227/neu.0000000000002961

**Published:** 2024-05-24

**Authors:** Milo Hollingworth, Lisa J. Woodhouse, Zhe K. Law, Azlinawati Ali, Kailash Krishnan, Robert A. Dineen, Hanne Christensen, Timothy J. England, Christine Roffe, David Werring, Nils Peters, Alfonso Ciccone, Thompson Robinson, Anna Członkowska, Dániel Bereczki, Juan José Egea-Guerrero, Serefnur Ozturk, Philip M. Bath, Nikola Sprigg

**Affiliations:** * Department of Neurosurgery, Nottingham University Hospitals, Nottingham, UK;; ‡ Stroke Trials Unit, Mental Health & Clinical Neurosciences, Queen's Medical Centre, School of Medicine, University of Nottingham, Nottingham, UK;; § Department of Medicine, Faculty of Medicine, National University of Malaysia, Kuala Lumpur, Malaysia;; ‖ Faculty of Health Sciences, School of Medical Imaging, University of Sultan Zainal Abidin, Kuala Nerus, Malaysia;; ¶ Stroke, Department of Acute Medicine, Nottingham University Hospitals, Nottingham, UK;; # Radiological Sciences, Mental Health and Clinical Neuroscience, University of Nottingham, Nottingham, UK;; ** NIHR Nottingham Biomedical Research Centre, Nottingham, UK;; †† Department of Neurology, Copenhagen University Hospital, Bispebjerg, Denmark;; ‡‡ Stroke, Royal Derby Hospital, University Hospitals of Derby and Burton, Derby, UK;; §§ Stroke Research, School of Medicine, Keele University, Newcastle under Lyme, UK;; ‖‖ Stroke Research Centre, Brain Repair & Rehabilitation, UCL Queen Square Institute of Neurology, London, UK;; ¶¶ Stroke Center and Department of Neurology, University Hospital Basel, University of Basel, Basel, Switzerland;; ## Azienda Socio Sanitaria Territoriale di Mantova, Mantova, Italy;; *** College of Life Sciences, University of Leicester, Leicester, UK;; ††† Institute of Psychiatry and Neurology, Warsaw, Poland;; ‡‡‡ Department of Neurology, Semmelweis University, Budapest, Hungary;; §§§ Unidad de NeuroCríticos Hospital Univ. Virgen del Rocío, Sevilla, Spain;; ‖‖‖ Department of Neurology, Neurointensive Care- Stroke Center, Selcuk University Faculty of Medicine, Konya, Turkey

**Keywords:** Tranexamic acid, Intracerebral hemorrhage, Neurosurgery, Stroke

## Abstract

**BACKGROUND AND OBJECTIVES:**

An important proportion of patients with spontaneous intracerebral hemorrhage (ICH) undergo neurosurgical intervention to reduce mass effect from large hematomas and control the complications of bleeding, including hematoma expansion and hydrocephalus. The Tranexamic acid (TXA) for hyperacute primary IntraCerebral Hemorrhage (TICH-2) trial demonstrated that tranexamic acid (TXA) reduces the risk of hematoma expansion. We hypothesized that TXA would reduce the frequency of surgery (primary outcome) and improve functional outcome at 90 days in surgically treated patients in the TICH-2 data set.

**METHODS:**

Participants enrolled in TICH-2 were randomized to placebo or TXA. Participants randomized to either TXA or placebo were analyzed for whether they received neurosurgery within 7 days and their characteristics, outcomes, hematoma volumes (HVs) were compared. Characteristics and outcomes of participants who received surgery were also compared with those who did not.

**RESULTS:**

Neurosurgery was performed in 5.2% of participants (121/2325), including craniotomy (57%), hematoma drainage (33%), and external ventricular drainage (21%). The number of patients receiving surgery who received TXA vs placebo were similar at 4.9% (57/1153) and 5.5% (64/1163), respectively (odds ratio [OR] 0.893; 95% CI 0.619-1.289; *P*-value = .545). TXA did not improve outcome compared with placebo in either surgically treated participants (OR 0.79; 95% CI 0.30-2.09; *P* = .64) or those undergoing hematoma evacuation by drainage or craniotomy (OR 1.19 95% 0.51-2.78; *P*-value = .69). Postoperative HV was not reduced by TXA (mean difference −8.97 95% CI −23.77, 5.82; *P*-value = .45).

**CONCLUSION:**

TXA was not associated with less neurosurgical intervention, reduced HV, or improved outcomes after surgery.

## Graphical abstract

**Figure:**
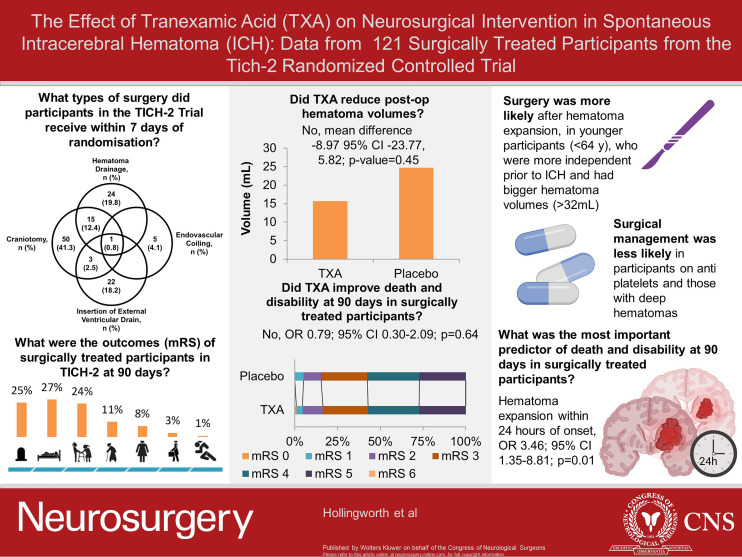

**ABBREVIATIONS:**
CRF: case report form
DWIHL: diffusion-weighted imaging lesions
HE: hematoma expansion
HV: hematoma volume
MD: mean difference
NIHSS: National Institute of Health Stroke Scale
RCT: randomized controlled trial
TICH-2: TXA in IntraCerebral Hemorrhage-2
TXA: tranexamic acid
VTE: venous thromboembolism.

Lifetime risk of intracerebral hemorrhage (ICH) is 8%.^1^ There are few effective treatments, and outcomes have failed to improve over time.^2^ Less than half of patients survive beyond 2 years—most dying within a month.^2,3^ Age, diabetes mellitus, impaired consciousness, hematoma volume (HV), intraventricular hemorrhage (IVH), infratentorial location, and small vessel disease predict early mortality, but hematoma expansion (HE) remains an important modifiable risk factor.^4-7^ Lowering blood pressure and correcting coagulopathy can reduce the risk of HE.^8,9^ Surgery can help reduce mass effect and/or intracranial pressure after ICH, immediately or after neurological deterioration. Exactly in whom and how surgery should be used remains controversial. Recently, the industry-funded Early Minimally Invasive Removal of Intracerebral Hemorrhage (ENRICH) trial reported that minimally invasive trans-sulcal parafascicular surgery improved outcomes for participants with lobar ICH within 24 hours of ictus.^10^ However, no other clinical trial has demonstrated that surgical intervention improves outcome.^11-15^ Nevertheless, pooled data support surgical intervention, and surgery is thought to be a reasonable intervention in patients with hydrocephalus and patients with Glasgow Coma Scale (GCS) 10 to 13, with hematomas that are large or centered in the cerebellum or temporal lobe.^16-19^

Tranexamic acid (TXA) is a lysine analog that prevents plasmin-mediated degradation of fibrin helping to stop hemorrhage. TXA has been studied in spontaneous ICH (summarized in Table 1), as well as other neurosurgical pathologies such as subarachnoid hemorrhage, acute head injury, craniosynostosis surgery, chronic subdural hematoma, meningioma surgery, and perinatal periventricular hemorrhage (summarized in **Supplemental Digital Content 1** [http://links.lww.com/NEU/E221]). The TXA in IntraCerebral Hemorrhage-2 (TICH-2), a randomized double-blind placebo-controlled trial, recruited 2325 participants and showed TXA reduced HE and early neurological deterioration.^25^ TXA may also reduce brain injury arising from neurotoxicity and neuro-inflammation.^27^ Therefore, TXA could reduce need for rescue surgery, reduce secondary brain injury, and possibly reduce intraoperative bleeding.

**TABLE 1.** Summary Table of Clinical Studies Using TXA in Spontaneous Intracerebral Hemorrhage

Reference	Population	Design/intervention	Comparators	Outcome	Notes
Polymeris et al,^20^ 2023	Patients with direct oral anticoagulant associated ICH within 12 h of symptom onset and 48 h of last NOAC Median age of participants 82 y N = 63; 6 centers; Switzerland.	Double-blind, RCT: IV TXA (1 g over 10 min followed by 1 g over 8 h) or matching placebo in addition to standard medical care.	Primary outcome was hematoma expansion, defined as ≥33% relative or ≥6 mL absolute volume increase at 24 h and analyzed using logistic regression adjusted for baseline hematoma volume on an intention-to-treat basis.	The primary outcome did not differ between TXA (n = 32) and placebo (n = 31). Between the TXA and placebo arms, the proportion of participants who died or had major thromboembolic complications within 90 d did not differ.	There was a signal for interaction with onset-to-treatment time (*P*-interaction = 0.024), favoring TXA when administered within 6 h of symptom onset. All thromboembolic events occurred at least 2 wk after study treatment, exclusively in participants not restarted on oral anticoagulation.
Pszczolkowski et al,^21^ 2022	TICH-2. Adults with acute spontaneous ICH, presenting within 8 h of ictus. N = 253; 23 centers; international.	RCT; TXA (IV 1 g bolus, 1 g infusion/8 h) or placebo.	Prevalence and number of remote DWIHLs were compared between the treatment groups using binary logistic regression adjusted for baseline covariates.	96 (43.8%) were randomized to receive TXA and 123 (56.2%) were randomized to receive placebo. There was no increase for the TXA group compared with the placebo group in DWIHL prevalence (20 of 96 [20.8%] vs 28 of 123 [22.8%]; OR, 0.71; 95% CI, 0.33-1.53; *P* = .39) or mean (SD) number of DWIHLs (1.75 [1.45] vs 1.81 [1.71]; MD, −0.08; 95% CI, −0.36 to 0.20; *P* = .59).	Participants who were randomized within 3 h of ICH onset or those with chronic infarcts appeared less likely to have DWIHLs if they received TXA. Participants with probable cerebral amyloid angiopathy appeared more likely to have DWIHLs if they received TXA.
Law et al,^22^ 2021	TICH-2. Adults with acute spontaneous ICH, presenting within 8 h of ictus. N = 2325; multicenter; International.	RCT; TXA (IV 1 g bolus, 1 g infusion/8 h) or placebo.	Neurological deterioration (increase NIHSS of ≥4 points or a decline in GCS of ≥2 from baseline assessed at day 2 [early] and day 7 [late]). Hematoma expansion defined as an increase in intraparenchymal hematoma volume on follow-up scan (at 24 h) of >33% or >6 mL from baseline volume.	TXA reduced neurological deterioration within 7 d (aOR 0.79, 95% CI 0.64-0.97; *P* = .026) and early (aOR 0.79, 95% CI 0.63-0.99; *P* = .041) but not late neurological deterioration TXA reduced the risk of hematoma expansion (aOR 0.76, 0.62-0.93; *P* = .008) and hematoma progression (aOR 0.71, 0.59-0.86; *P* < .001) but not edema growth at 24 h.	
Ovesen et al, 2021^23^	TICH2. Spot-sign positive spontaneous ICH in adults presenting to hospital within 8 h participating in the TICH-2 trial. N = 215; multicenter; international.	RCT; TXA (IV 1 g bolus, 1 g infusion/8 h) or placebo.	Primary outcome was absolute day-2 intraparenchymal hematoma volume and day-2 intraparenchymal and intraventricular hematoma volume.	Presence of a spot sign did not modify the treatment effect of TXA vs placebo.	There was low statistical power and treatment delay in participants receiving CT angiography.
Meretoja et al,^24^ 2020	Patients >18 y with a spot sign, >4.5 h of symptom onset, excluding massive hematomas and moribund patients. N = 100; 12 centers; Australia, Finland, and Taiwan.	RCT; TXA 1 g in 100 mL 0.9% NaCl over 10 min followed by 1 g over 8 h or placebo started within 4.5 h of symptom onset.	The primary outcome was intracerebral hemorrhage growth (>33% relative or >6 mL absolute) at 24 h. The primary and safety analyses were done in the intention-to-treat population.	No evidence that TXA prevents intracerebral hemorrhage growth, although the treatment was safe with no increase in thromboembolic complications.	
Sprigg et al,^25^ 2018	TICH-2. Adult patients (aged ≥18 y) with ICH within 8 h of onset excluding secondary ICH (anticoagulation, thrombolysis, trauma, known underlying structural abnormality) prestroke dependence (mRS score >4), life expectancy <3 mo or GCS <5. N = 2307; multicenter; Denmark, Georgia, Hungary, Ireland, Italy, Malaysia, Poland, Spain, Sweden, Switzerland, Turkey, and The United Kingdom (majority).	Double blind RCT; 1 g of an IV TXA bolus followed by an 8 h 1 g infusion or matching placebo (i.e. 0.9% saline).	The primary outcome was functional status (death or dependency) at day 90, measured by mRS score, using ordinal logistic regression, with adjustment for stratification and minimisation criteria.	TXA, n = 1152; placebo, n = 1155. There was no statistically significant difference in functional status at day 90 [adjusted OR 0.88, 95% CI 0.76 to 1.03; *P* = .11]. There were fewer deaths by day 7 in the TXA group but no difference in case fatality at 90 d.	Fewer patients experienced serious adverse events after treatment with TXA than with placebo by days 2 and 7 (there was no increase in thromboembolic events or seizures).
Sprigg et al,^26^ 2014	Adults, excluding secondary ICH (anticoagulation, known vascular malformations) and those with previous vascular occlusive events or VTE N = 24; single centre; The United Kingdom.	RCT; TXA (IV 1 g bolus, 1 g infusion/8 h) or placebo within <24 h of ictus.	Trial feasibility (primary); secondary thromboembolic events; functional outcome; NIHSS; hematoma growth.	16 (TXA) vs 8 participants; trial was feasible; no differences in secondary outcomes.	

CT, computed tomography; DWIHL, diffusion-weighted imaging lesions; GCS, Glasgow Coma Scale; IV, intravenous; MD, mean difference; mRS, modified Rankin Score; NIHSS, National Institute of Health Stroke Scale; OR, odds ratio; RCT, randomized controlled trial; TXA, tranexamic acid; TICH-2, TXA in IntraCerebral Hemorrhage-2; VTE, venous thromboembolism.

We hypothesized that TXA would reduce surgical intervention after ICH, improve outcome for surgical participants, and reduce postoperative HVs. We analyzed data from the TICH-2 trial to test these hypotheses and also to describe the characteristics of surgically treated participants.

## METHODS

**FIGURE 1.**
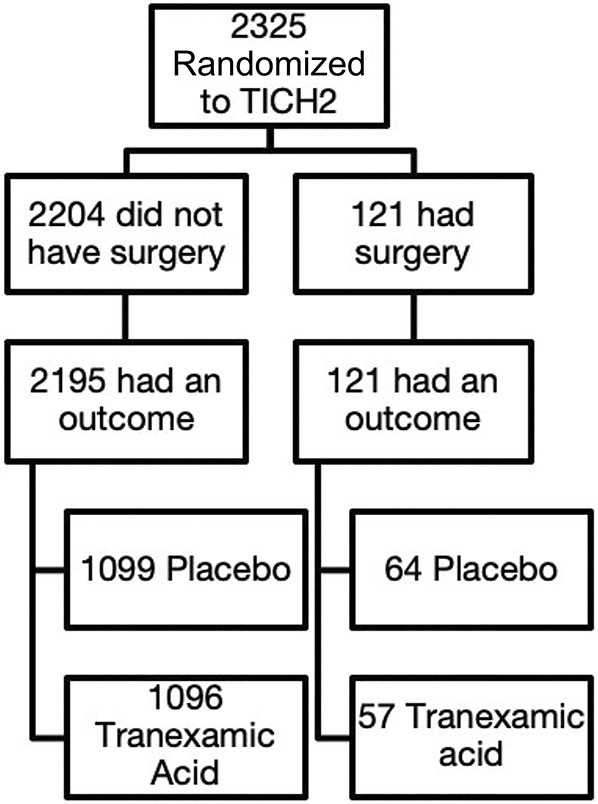
Post hoc analysis of surgically treated spontaneous intracerebral hematoma from the TICH-2 trial. TICH-2, Tranexamic acid in IntraCerebral Hemorrhage-2.

This is post hoc analysis of data from the TICH-2 trial (ISRCTN93732214). The TICH-2 protocol has been published in detail (Figure 1).^28^ No ethical approval for this analysis of fully anonymized data. The TICH-2 trial was approved by Medicines and Healthcare products Regulatory Agency and received approval by the National Research Ethics Service Committee East Midlands 23/11/2012, ref: 12/EM/0369. In brief, the TICH-2 trial was designed to measure the efficacy of TXA as an off-label investigational product to reduce death and dependency at 90 days after spontaneous ICH, measured by ordinal shift analysis of the modified Rankin Scale (mRS) (0-6). Consent was acquired from patients, relative proxies (or independent doctors when necessary) presenting within 8 hours of spontaneous ICH.^25^ Participants were randomized to receive intravenous TXA as a 1 g loading dose followed by a further infusion of 1 g over 8 hours or placebo, using a random allocation sequence generated using the trial packaging. Key eligibility criteria stipulated participants were adults with acute ICH admitted to a participating hospital within 8 h of stroke symptom onset (or time last seen well). Participants who underwent immediate surgery; had ICH secondary to anticoagulation, trauma, known structural abnormalities; prestroke dependence (mRS >4); life expectancy <3 months; and GCS <5 were excluded. Routine care such as blood pressure lowering treatment, neurosurgery, and venous thromboembolism prophylaxis was recorded prospectively and provided at the discretion of local care providers.

In this analysis, our primary outcome was surgery performed within 7 days, which was documented on the case report form (CRF). Surgical intervention was divided into placement of external ventricular drain, craniotomy, minimally invasive drainage procedures, or endovascular coiling. Although, participants who underwent multiple procedures were counted as surgically managed participants, further details of reoperation (ie, indication, timing, procedure order) were not recorded on the CRF. Date of surgery was determined as the date of first intervention. Characteristics and outcomes of surgically treated participants who received TXA were compared with placebo. HE was defined as an absolute increase of more than 6 mL or a relative growth of >33% between baseline imaging and repeat imaging performed at 24 hours. Neurological deterioration was defined as an increase in National Institute of Health Stroke Scale by 4 points or more or reduction in GCS by 2 or more points. HV on preoperative imaging and postoperative HV were compared if, first, a postoperative computed tomography scan was performed and, second, if the participant had received hematoma evacuation (by either drainage or craniotomy). Participants who did not have a postoperative scan or did not receive hematoma evacuation were excluded from volumetric analysis. Hematoma and perihematoma edema volumes were calculated by segmentation using ITK Snap (Version 3.8.0) as previously described.^29,30^ Hematoma clearance was defined as the difference in HV between the preoperative and postoperative imaging. Time to surgery was calculated in days from date of ictus to date of surgery. For those with a postoperative scan, time to surgery was also estimated based on time from ictus to postoperative imaging (if the interval on the CRF was longer).

### Statistical Analysis

Participant characteristics were compared between those who had neurosurgery vs those who did not. To identify independent predictors of surgical intervention, variables that were significantly different following univariate analysis (*P*-value <.05) were carried forward into a predictive model and interrogated using step-backward regression—removing nonsignificant variables until only significant variables remained. Similarly, independent predictors of achieving unfavorable outcomes (mRS 4-6) at 90 days in surgically treated participants were identified using the same step-backward approach until only independent predictors of outcome remained.

Statistical analysis was performed using SPSS version 23.0 (IBM). Normally distributed values were described in mean, mean difference (MD), and SD, and non-normally distributed data were described using median and IQR. Univariate analysis was performed using *t*-test continuous variables. Dichotomous variables were compared using binary logistic regression. Continuous variables were dichotomized at the midpoint between surgical and nonsurgical groups: time to randomization (≤3.75 and >3.75 hours) and HV (≤32 mL and >32 mL). The primary outcome of this analysis was surgical intervention. Secondary outcomes were postoperative HV, hematoma clearance, early death (day 7), and function at 90 days (mRS, cognition, quality of life, and depression). Comparison of continuous variables used the Student *t*-test and reported as MD binary outcomes were reported as odds ratio (OR). Statistical significance was defined as *P*-value<.05.

## RESULTS

**FIGURE 2.**
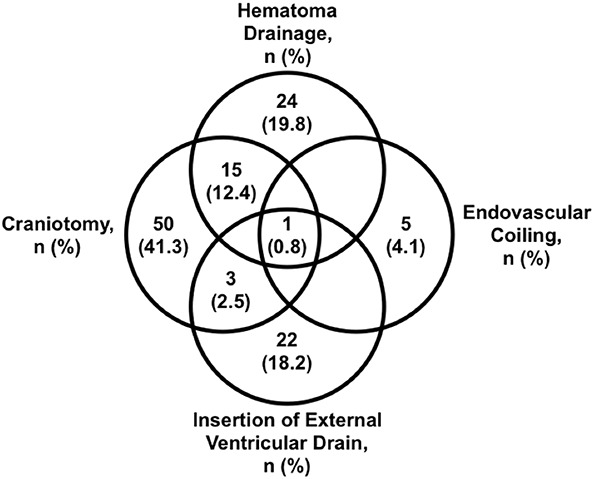
Venn diagram of types of surgical/radiological intervention performed within 7 d of 121 surgically treated intracerebral hematoma patients in the TICH-2 trial. An additional patient received an external ventricular drain and hematoma drainage. TICH-2, Tranexamic acid in IntraCerebral Hemorrhage-2.

Of the 2325 participants who were randomized in TICH-2, 121 underwent neurosurgical intervention (Figure 2) including craniotomy (69/121; 57%), hematoma drainage (26/121; 33%) and external ventricular drainage (46/121; 21%). Six participants had an underlying cerebral aneurysm and received endovascular coiling. Nineteen participants received more than 1 surgical procedure. Median time from onset to surgery was 1 day (IQR 2 days).

Participants who had surgery were younger (61.5 [SD 11.9] vs 69.3 years [SD 13.8]; MD −7.86 years; 95% CI −10.37, −5.35; *P* = .03) and had better premorbid functional status (mRS, 1 vs 0.57; *P* = .002), but also had a lower GCS (12.6 vs 13.5; MD −0.87; 95% CI −1.26 to −0.48; *P* < .001) and larger HV (41.3 mL vs 23.0 mL; MD 18.4 mL; 95% CI 13.42-23.29; *P* = .004, Table 2). There was no difference in patients with venous thromboembolism or those on high blood pressure treatment. Lobar and infratentorial hematomas were more common among surgically treated participants at 43.8% vs 31.1% (OR 1.75; 95% CI 1.21 to 2.53; *P* = .004) and 11% vs 6% (OR 1.97; 95% CI 1.10-3.53; *P* = .02), respectively. Deep supratentorial hematomas were more common in nonsurgically managed participants (42.1 vs 59.8%; OR 0.49; 95% CI 0.34, 0.71; *P* < .001). Surgically treated participants were more likely to have HE within 24 hours (OR 2.45; 95% CI 1.68-3.56; *P* < .001). Significant differences identified from the univariate analysis demonstrated that age ≤64 years, greater time to randomization, premorbid mRS <1, IVH, GCS <13, greater HV, and presence of HE were more likely to have surgical intervention (Table 2). Participants taking prior antiplatelet therapy were less likely to have surgical intervention. After step-backward regression, HE (OR 1.20; 95% CI 1.33-2.93; *P* < .001), younger age <64 years (OR 2.89; 1.92-4.36; *P* < .001), HV > 32 mL (OR 4.86; 3.24-7.27; *P* < .001), and better prefunctional status (OR 2.57; 1.48-4.47; *P* < .001) independently predicted surgical intervention.

**TABLE 2.** Baseline Characteristics of Patients Treated Surgically and Nonsurgically in the TICH-2 Trial

Characteristic	Neurosurgery	No neurosurgery	OR/MD (95% CI)	*P*-value
n, (%)	121 (5.20)	2195 (94.20)		
Age, mean (SD)	61.46 (11.99)	69.32 (13.79)	−7.86 (−10.37, −5.35)	.03
Sex, male (%)	74 (61.10)	1223 (55.70)	1.25 (0.86, 1.81)	.26
Time from onset to randomization, mean (SD)	3.49 (1.44)	3.96 (1.71)	−0.47 (−0.74, −0.20)	.02
Days from onset to surgery, mean (SD)	1.08 (1.38)	—	—	—
Prestroke modified Rankin Scale, mean (SD)	0.27 (0.82)	0.57 (1.03)	−0.29 (−0.48, −0.11)	.002
Admission NIHSS, mean (SD)	14.70 (7.74)	12.91 (7.44)	−1.79 (−3.15, −0.42)	.013
Admission Glasgow Coma Scale, mean (SD)	12.62 (2.57)	13.49 (2.10)	−0.87 (−1.26, −0.48)	<.001
Intraventricular hemorrhage, yes (%)	58 (47.93)	686 (31.25)	2.03 (1.40, 2.93)	<.001
History of stroke or TIA, yes (%)	14 (11.57)	314 (14.30)	1.03 (0.56, 1.91)	.56
History of antiplatelets, yes (%)	21 (17.50)	586 (26.69)	0.54 (0.32, 0.91)	.03
Blood pressure treatment, yes (%)	100 (84.75)	1819 (83.06)	1.31 (0.77, 2.23)	.97
History of venous thromboembolism, yes (%)	3 (2.47)	31 (1.41)	1.92 (0.57, 6.47)	.29
Hematoma location				
Supratentorial lobar, n (%)	53 (43.80)	684 (31.16)	1.75 (1.21, 2.53)	.004
Supratentorial deep, n (%)	51 (42.14)	1313 (59.81)	0.49 (0.34, 0.71)	<.001
Infratentorial, n (%)	14 (11.57)	134 (6.10)	1.97 (1.10, 3.53)	.02
Combination, n (%)	3 (2.47)	64 (5.64)	0.85 (0.26, 2.73)	.78
Baseline hematoma volume (mL), mean (SD)	41.32 (30.11)	22.96 (26.60)	18.36 (13.42, 23.29)	.004
TXA administered, n (%)	57 (47.11)	1096 (49.93)	0.893 (0.619, 1.289)	.545
Hematoma expansion, yes (%)	52 (42.97)	516 (23.50)	2.45 (1.68, 3.56)	<.001

MD, mean difference; NIHSS, National Institute of Health Stroke Scale; OR, odds ratio; TIA, transient ischemic attack; TXA, tranexamic acid; TICH-2, TXA in IntraCerebral Hemorrhage-2.

Outcomes were compared between surgically treated and nonsurgically treated participants, adjusting for baseline characteristics (age, mRS, National Institute of Health Stroke Scale, HV, and IVH) (Table 3). Participants who were treated with neurosurgery were less likely to have died by day 7 (OR 0.15, 95% CI 0.06-0.40; *P* < .001); however, this was not found at day 90 (OR 1.11; 95% CI 0.63-1.95; *P* = .77; Table 3). There was no significant difference in safety outcomes between surgically and nonsurgically treated participants except for seizures, which were more common in surgically treated participants (15.7% vs 6.5%; OR 2.69; 95% CI 1.60-4.52; *P* = .001) (**Supplemental Digital Content 2** [http://links.lww.com/NEU/E222]) There was no significant difference in venous thromboembolism, which occurred in 5% of surgically participants and 3.1% in medically managed participants. The characteristics of surgical participants who had favorable (mRS 0-3) and unfavorable outcomes (mRS 4-6) were compared (Table 4). Surgical participants were more likely to have an unfavorable outcome (mRS 4-6) at 90 days (OR 3.34; 95% CI 1.80-6.01; *P* = .008). Death and severe disability (mRS 5 or 6) at 90 days were also more likely in surgically treated participants (OR 2.19; 95% CI 1.22-3.92; *P* = .01) (Table 5). Deep supratentorial hematomas and HE were less common in those with favorable outcomes OR 0.54 (95% CI 0.20-0.48; *P* = .02) and OR 0.30 (95% CI 0.12-0.77 *P* = .01), respectively. Only HE within 24 hours of randomization independently predicted unfavorable outcome in surgically treated participants (OR 3.46; 95% CI 1.35-8.81; *P* = .01). Days to surgery were less for participants who had an unfavorable outcome (MD −0.63, 95% CI −1.22 to 0.058; *P* = .031).

**TABLE 3.** Outcomes of Patients Treated Surgically and Nonsurgically in the TICH-2 Trial

	Neurosurgery	No neurosurgery	OR/MD (95% CI)	*P*-value
Day 7				
Glasgow Coma Scale, mean (SD)	10.29 (3.93)	13.56 (2.68)	MD −3.06 (−3.53, −2.59)	<.001
NIHSS, mean (SD)	18.60 (11.73)	9.90 (8.14)	OR 6.88 (5.50, 8.25)	<.001
Do not attempt resuscitation, yes (%)	13 (10.74)	494 (22.51)	OR 0.58 (0.30, 1.16)	.12
Intensive care unit transfer, yes (%)	108 (89.26)	124 (5.65)	OR 140.91 (74.19, 267.71)	<.001
Invasive ventilation, yes (%)	90 (74.38)	76 (3.46)	OR 84.33 (49.75, 143.01)	<.001
Neurological deterioration, n (%)	94 (77.69%)	585 (26.65%)	OR^a^ 16.39 (9.98, 26.92)	<.001
Deaths by day 7, n (%)	6 (4.96)	217 (9.89)	OR^a^ 0.15 (0.06, 0.40)	<.001
Day 90				
Total deaths, n (%)	30 (24.79)	473 (21.55)	OR^a^ 1.11 (0.63, 1.95)	.77
mRS, mean (SD)	4.37 (1.43)	3.65 (1.74)	MD −0.72 (−1.03, −0.401)	<.001
Favorable outcome (mRS 0-4)			OR^a^ 3.34 (1.80, 6.01)	.008
Dead or severely disabled (mRS 5 or 6)			OR^a^ 2.19 (1.22, 3.92)	.01
Barthel Index, mean (SD)	49.65 (35.86)	70.76 (34.71)	MD −17.4 (−23.2, −11.6)	<.001
EuroQol5D, mean (SD)	0.17 (0.34)	0.35 (0.40)	MD −0.20 (−0.26, −0.14)	<.001
Cognition, mean (SD)	24.68 (4.19)	23.39 (5.43)	MD 1.13 (−1.16, 3.43)	.33
Zung Depression Scale, mean (SD)	45.34 (14.62)	45.84 (14.21)	MD −1.43 (−7.33, 4.46)	.63

MD, mean difference; mRS, modified Rankin Scale; NIHSS, National Institute of Health Stroke Scale; OR, odds ratio; TICH-2, Tranexamic acid in IntraCerebral Hemorrhage-2.

a Outcomes adjusted for baseline age, mRS, NIHSS, baseline hematoma volume and intraventricular hemorrhage.

**TABLE 4.** Outcomes of Surgically Treated Participants From the TICH-2 Trial

	Favorable outcome mRS 0-3	Unfavorable outcome mRS 4-6	OR/MD (95% CI)	*P*-value
N, (%)	28 (23.14)	93 (76.86)		<.001
Age, mean (SD)	60.32 (11.08)	61.47 (12.34)	MD 1.41 (−3.52 to 6.36)	.59
Sex, male (%)	17 (60.71)	57 (61.95)	OR 0.94 (0.39 to 2.26)	.91
Time from onset to randomization, mean (SD)	3.41 (1.43)	3.77 (1.49)	MD −0.36 (−0.98 to 0.26)	.25
Days to surgery, mean (SD)	1.57 (1.68)	0.93 (1.25)	MD −0.63 (−1.22 to −0.058)	.031
mRS, mean (SD)	0.11 (0.41)	0.33 (0.90)	MD 0.21 (−0.13 to 0.57)	.22
Glasgow Coma Scale, mean (SD)	13.32 (2.16)	12.38 (2.66)	MD −0.94 (−2.03 to 0.15)	.09
Intraventricular hemorrhage, yes (%)	10 (35.71)	47 (51.08)	OR 0.53 (0.22 to 1.28)	.15
History of antiplatelets, yes (%)	5 (17.85)	16 (17.39)	OR 1.03 (0.34 to 3.13)	.96
History of stroke or TIA, yes (%)	2 (7.14)	12 (13.04)	OR 0.42 (0.91 to 2.01)	.49
History of venous thromboembolism, yes (%)	0 (0)	3 (3.26)	OR 0.64 (0.72 to 5.76)	.58
Hematoma location				
Supratentorial lobar, n (%)	16 (57.14)	36 (42.39)	OR 2.07 (0.88 to 4.89)	.09
Supratentorial deep, n (%)	7 (25.00)	44 (47.82)	OR 0.54 (0.20 to 0.48)	.03
Infratentorial, n (%)	5 (17.86)	9 (1.09)	OR 2.01 (0.61 to 6.57)	.24
Combination, n (%)	0 (0)	3 (3.26)	OR 0.45 (0.02 to 8.95)	.33
Baseline HV (mL), mean (SD)	33.24 (30.79)	43.78 (30.79)	MD 10.53 (−2.25 to 23.31)	.11
Baseline PHE volume (mL), mean (SD)	15.97 (15.44)	21.67 (20.13)	MD 5.70 (−2.42 to 13.83)	.16
Baseline intraventricular HV (mL), mean (SD)	14.80 (14.02)	14.38 (15.35)	MD −0.41 (−9.42 to 8.59)	.93
Hematoma expansion, yes (%)	6 (28.60)	46 (56.50)	OR 0.30 (0.12 to 0.77)	.01

HV, hematoma volume; MD, mean difference; mRS, modified Rankin Scale; OR odds ratio; PHE, perihematoma edema; TIA transient ischemic attack; TICH-2, Tranexamic acid in IntraCerebral Hemorrhage-2.

**TABLE 5.** TXA vs Placebo in Participants Receiving Surgery Within 7 Days of Ictus During the TICH-2 Trial

	Tranexamic acid (n = 57)	Placebo (n = 64)	OR/MD (95% CI)	*P*-value
Neurosurgical intervention				
Craniotomy, n (%)	31 (54.30)	38 (59.30)	OR 0.81 (0.39 to 1.68)	.58
Hematoma drainage, n (%)	25 (43.90)	21 (32.80)	OR 0.95 (0.45 to 2.03)	.90
External ventricular drain, n (%)	13 (22.80)	13 (20.30)	OR 1.15 (0.49 to 2.76)	.74
Endovascular coiling, n (%)	1 (1.70)	5 (7.80)	OR 0.21 (0.24 to 1.86)	.13
Age, mean (SD)	61.65 (11.94)	61.30 (12.14)	MD −0.35 (−4.69 to 3.99)	.77
Days from onset to surgery, mean (SD)	0.77 (0.96)	1.36 (1.60)	MD 0.59 (0.09 to 1.08)	.06
Sex, male (%)	34 (59.60)	40 (62.50)	OR 0.88 (0.43 to 1.85)	.75
Time from onset to randomization, mean (SD)	3.47 (1.40)	3.52 (1.49)	MD 0.04 (−0.48 to 0.57)	.90
mRS, mean (SD)	0.28 (0.81)	0.27 (0.82)	MD −0.15 (−0.31 to 0.28)	.94
GCS, mean (SD)	12.37 (2.82)	12.84 (2.32)	MD 0.47 (−0.45 to 1.40)	.49
Intraventricular hemorrhage, yes (%)	33 (57.89)	25 (39.06)	OR 2.14 (1.04 to 4.44)	.04
History of previous antiplatelets, yes (%)	11 (19.30)	10 (15.63)	OR 1.29 (0.50 to 3.31)	.59
Hematoma location				
Supratentorial lobar, n (%)	23 (40.35)	30 (46.88)	OR 0.76 (0.73 to 1.58)	.47
Supratentorial deep, n (%)	28 (49.12)	23 (35.94)	OR 1.72 (0.83 to 3.57)	.14
Infratentorial, n (%)	4 (7.02)	10 (15.63)	OR 0.40 (0.12 to 1.38)	.14
Combination, n (%)	1 (1.75)	2 (3.13)	OR 2.29 (0.20 to 25.96)	.49
Outcomes at 90 d				
Favorable outcomes (mRS 0-3), n (%)	14 (24.56)	14 (21.88)	OR 1.19 (0.51 to 2.78)	.69
Total deaths, n (%)	14 (24.56)	16 (25.00)	OR 0.97 (0.43 to 2.23)	.96
Barthel Index, mean (SD)	34.52 (39.44)	36.11 (39.09)	MD 1.59 (−12.99 to 16.18)	.83
EuroQol5D, mean (SD)	0.20 (0.33)	0.14 (0.33)	MD −0.06 (−0.19 to 0.06)	.64
Cognition, mean (SD)	9.96 (13.40)	8.00 (12.59)	MD −1.95 (−9.43 to 5.52)	.51
Subgroup analysis of participants undergoing hematoma evacuation by drainage or craniotomy with postoperative imaging				
Participants with postevacuation scan, n (%)	21 (36.80)	27 (42.10)	OR 0.38 (0.38 to 1.66)	.55
Preoperative volume (mL), mean (SD)	71.05 (32.60)	69.66 (39.51)	MD 1.38 (−19.91 to 22.68)	.89
Postoperative HV (mL), mean (SD)	15.71 (27.27)	24.68 (23.66)	MD −8.97 (−23.77 to 5.82)	.23
Haematoma evacuation (mL), mean (SD)	55.33 (33.79)	44.97 (48.43)	MD 10.36 (−14.35 to 35.07)	.40
Postoperative HV ≤15 mL, n (%)	16 (76.19)	13 (48.14)	OR 2.67 (0.79 to 8.97)	.13
Days from onset to surgery, mean (SD)	0.50 (0.67)	0.65 (0.89)	MD 0.22 (−0.89 to 1.35)	.66

HV, hematoma volume; GCS, Glasgow Coma Scale; MD, mean difference; mRS, modified Rankin Scale; OR, odds ratio; TXA, tranexamic acid; TICH-2, TXA in IntraCerebral Hemorrhage-2; TIA, transient ischemic attack; VTE, venous thromboembolism.

**FIGURE 3.**
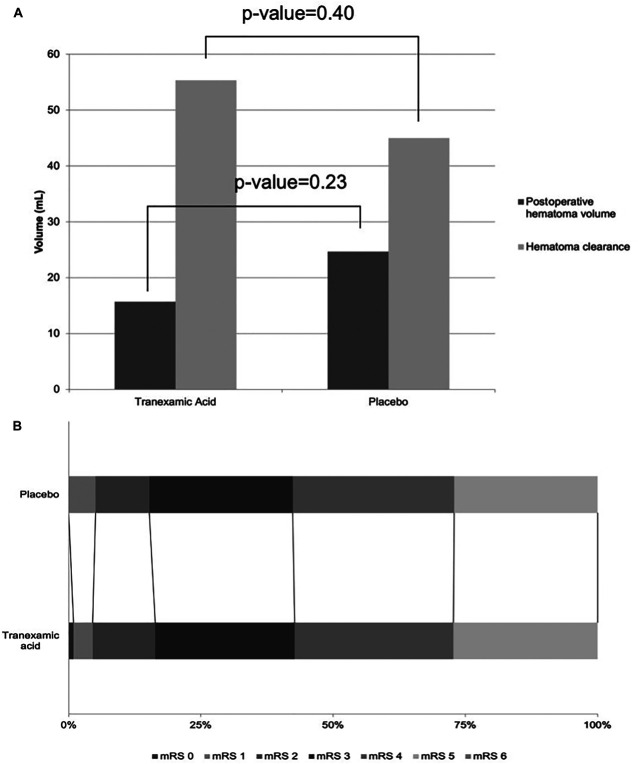
Outcomes of surgically participants of the TICH-2 trial who received TXA vs placebo. **A**, Postoperative intracerebral hematoma volume and hematoma evacuation was not significantly different between participants who received TXA compared with placebo. **B**, Functional outcome did not differ between patients who underwent tranexamic or placebo. mRS, modified Rankin Scale; TXA, tranexamic acid; TICH-2, TXA in IntraCerebral Hemorrhage-2.

There was no difference in the number of surgically treated participants who received TXA vs placebo, 57/1153 vs 64/1163, respectively (OR 0.893; 95% CI 0.619-1.289; *P*-value = .545) (Table 5). There was no significant difference in timing or types of surgery received between the TXA and placebo groups. Baseline IVH was more common in the TXA group (OR 2.14; 1.04-4.44; *P* = .04). Otherwise, TXA and placebo groups were well matched with no significant differences in age, premorbid mRS, GCS, history of stroke, transient ischemic attack, venous thromboembolism, previous antiplatelets, or hematoma location. At 90 days, total number of deaths, functional outcome, quality of life, cognition, or depression did not differ between TXA vs placebo (Table 5; Figure 3). Of those who received hematoma evacuation, either by craniotomy or hematoma drainage (95/2316), there was also no association between improved outcome and TXA (OR 0.79; 95% CI 0.30-2.09; *P* = .64). To ensure there were no differences were observed in participants receiving EVD and endovascular coiling, analysis was repeated returning nonsignificant of ORs 0.99 (95% CI 0.45-2.14; *P* = .982) and 4.9 (95% CI 0.58-42.64; *P* = .143), respectively. Equally, when cases receiving endovascular coiling were removed, no difference in surgical intervention was observed between participants who received TXA vs placebo, OR 1.05 (95% CI 0.72-1.52; *P* = .796). Secondary outcomes including total deaths, measures of cognition, depression, and quality of life did not differ between TXA and placebo.

Forty-eight of 95 (51.1%) participants who received hematoma evacuation, either by craniotomy or drainage, had a postoperative scan we could analyze. In this subgroup, the average time to evacuation was 0.5 days for TXA and 0.65 days for placebo (MD 0.22 days; 95% CI −0.89 to 1.35; *P* = .66). Postoperative hematomas were nonsignificantly smaller in the TXA group (15.7 vs 24.7 mL; MD −8.97 mL; −23.77 to 5.82; *P* = .23, Figure 3). Hematoma clearance was nonsignificantly higher in TXA group compared with placebo (55.3 vs 45.0 mL, MD 10.36; 95% CI −14.35 to 35.07; *P* = .23). Sixteen participants who received TXA had a postoperative residual hematoma of ≤15 mL compared with 13 who received placebo (OR 2.67; 95% CI 0.79-8.97; *P* = .40).

## DISCUSSION

We report the findings of surgically treated participants from the TICH-2 trial. TXA did not reduce the number of participants receiving surgery within 7 days after symptom onset, postoperative HVs, or improve their outcomes.

Studies have previously demonstrated reduced intraoperative bleeding with TXA after traumatic brain injury, crainiosynotosis surgery, and meningioma surgery.^31-36^ (**Supplemental Digital Content 1** [http://links.lww.com/NEU/E221]); however, to our knowledge, no such study has reported the specific outcomes in surgically treated patients in detail. One phase II trial demonstrated no effect of recombinant activated factor VII on postoperative HV in 21 patients with ICH.^37^ The strengths of this analysis include near-complete participant follow-up, comprehensive outcome measurement, and multicenter design. Although, surgery was not the primary focus of TICH-2, these data likely reflect real-world experience of many patients undergoing ICH surgery.

TICH-2 demonstrated that TXA was safe and reduced serious adverse events in patients with ICH.^25^ Because TXA reduces fibrinolysis, there are understandable concerns regarding thrombo-embolic complications. TXA has been extensively studied in subarachnoid hemorrhage, as means of reducing aneurysmal rebleed (**Supplemental Digital Content 1** [http://links.lww.com/NEU/E221]). Some studies demonstrated lower incidence of rebleed, but TXA had increased rates of hydrocephalus^38^ and cerebral infarction.^39,40^ In these older studies, doses of TXA were prolonged (up to 6 weeks) and at higher doses (up to 9 g/day).^39,41^ Studies using smaller doses for shorter durations very soon after injury did not demonstrate additional serious adverse events in subarachnoid hemorrhage,^42^ traumatic brain injury,^43^ or ICH^25^ (**Digital Supplementary Content 1** [http://links.lww.com/NEU/E221]). Nevertheless, later administration is associated with increased risk of thrombotic complications.^44^ In our cohort, there was no evidence surgically treated participants suffered more adverse events with TXA compared with placebo. Greater numbers of seizures were observed among surgically treated patients; however, this analysis is likely underpowered to address this question.

HE was independently predictive of surgical intervention. However, HE also predicted poor outcome. Surgery is thought to be a reasonable option in a subset of patients who deteriorate. However, our data suggest that surgery after expansion may be too late to salvage functional outcome. Pooled analysis of 2186 patients suggests clot evacuation is of benefit if undertaken early.^16^ These data suggest that the goal of surgery may not just be to remove toxic blood products and reduce mass effect but also to prevent the deleterious effects of significant HE. We suggest that those at high risk of HE may stand to benefit the most from early surgical evacuation, but this would need to be explored in a randomized trial.

It was notable that surgery was predictive of poor outcome after adjustment for major prognostic factors. It is clear that patients who have surgery have larger HV and more severe strokes; however, it will be important to identify what unmeasured variables may also contribute to the poorer outcomes of surgically treated patients. Without a matched cohort or a randomized trial, we cannot make authoritative conclusions about the impact of surgery on outcome. Promising reports of the ENRICH trial^10^ suggest minimally invasive trans-sulcal parafascicular surgery for patients may help improve outcomes, but it is evident that further trials are necessary. In this study, several neurosurgical techniques were used, reflecting the ongoing role of surgery for ICH and the multitude of unanswered questions regarding its role. There are several ongoing trials exploring the role of surgery in ICH including novel aspiration devices (INVEST: NCT02654015; MIND: NCT03342664), endoscopic drainage (NCT04805177), stereotactic aspiration (SOITBRE: NCT03957707; NCT04686877), ultra-early intervention (EVACUATE: NCT04434807), and decompressive craniectomy (SWITCH: NCT02258919).

Data from the minimally invasive surgery with thrombolysis in intracerebral hemorrhage evacuation III study^45^ suggest that achieving a good outcome after surgery requires small postoperative HV. It may be that future trials, with an appropriately identified patient group (ie, patients at high risk of HE) and a clearly defined surgical objective (ie, to clear the hematoma to ≤15 mL), may finally provide clarity on the role of surgery in ICH. If optimal hematoma evacuation is a pre-requisite for better outcomes after ICH, hemostatic agents may have an important role to play.

### Limitations

This post hoc analysis uses data to address a question that the trial was not originally designed to answer. The number of surgically treated participants was small and may not be generalizable to all patients with ICH, especially those receiving immediate surgery and those with very severe ICH. Even if TXA has an impact on surgical outcomes, this study is probably underpowered to address it. Indeed, surgical management was not a major focus of the TICH-2 trial; therefore, there are factors in the design and conduct of the trial that limit the granularity required examine the question definitively. The median time to surgery was 1.08 days as such the therapeutic effect of TXA, which has a half-life of 2 to 11 hours in many participants would have been significantly diminished by the time they received surgery. However, the participants for whom volumetric analysis could be completed had a shorter time to surgery, as such TXA may still have been active in some patients. Nevertheless, there was no difference in hematoma clearance or postoperative HV. Decisions about surgical intervention were not standardized, and we cannot guarantee patients had equal access to surgical intervention. Equally randomization may have affected decision making. Sixteen percent of participants underwent multiple procedures, and the interaction of different procedures with outcomes and TXA cannot be deduced. Postoperative imaging was also not mandated and so HV could not be measured for all relevant participants, and indeed, imaging may have been performed on a clinical basis, which may bias the subgroup toward patients with complications, or those who deteriorated. Exact time from ictus to surgery could not be measured and as such temporal relationships between surgical intervention, early neurological deterioration, HE, and administration of TXA cannot be precisely studied. There are also no details of other factors related to surgery, such as use of intracranial pressure monitoring, accuracy of EVD placement, and incidence of abscess, meningitis, and ventriculitis, redo surgery, and postsurgical HE.

## CONCLUSION

In this post hoc analysis of surgically treated patients from the TICH-2 trial, we found no evidence that TXA given within 8 hours reduced surgery or improved outcome of surgically treated patients or postoperative HV. Earlier administration within 4.5 hours could potentially be more effective in preventing deterioration and the need for surgery. The ongoing TICH-3 trial of TXA (ISRCTN97693550) will examine this question in a larger cohort.

## Supplementary Material

SUPPLEMENTARY MATERIAL

## Notes

**Supplemental Digital Content 1. Table 1.** Population, intervention/design, comparators, and outcomes of clinical studies of tranexamic acid in cranial pathology identified from MEDLINE. AKI, acute kidney injury; AVM, arteriovenous malformation; CPR, cardiorespriatory resuscitation; CSF, cerebrospinal fluid; CT, computed tomography; DVT, deep vein thrombosis; GCS, Glasgow Coma Scale; GOS, Glasgow Outcome Score; GOSE, Glasgow Outcome Score Extended; ICH, intracerebral hemorrhage; ICU, intensive care unit; IV, intravenous; LP, lumbar puncture; MRI, magnetic resonance imaging; mRS modified Rankin Score; RCT, randomized controlled trial; SBP, systolic blood pressure; TBI, traumatic brain injury; TCD, transcranial Doppler ultrasound; VTE, venous thromboembolism. **Supplemental Digital Content 2**. **Table 2**. Safety outcomes at 90 days in patients treated surgically and nonsurgically in the TICH-2 trial.
