# Supplementary material for: The Effect of Tranexamic Acid on Neurosurgical Intervention in Spontaneous Intracerebral Hematoma: Data From 121 Surgically Treated Participants From the Tranexamic Acid in IntraCerebral Hemorrhage-2 Randomized Controlled Trial
Source: Neurosurgery. 2024 May 24;95(3):605–16. doi: 10.1227/neu.0000000000002961 (PMC11302947; doi:10.1227/neu.0000000000002961)
Supplement: SUPPLEMENTARY MATERIAL [file neu-95-605-s001.docx]

| Supplementary Material 1. Summary Table of Clinical Studies exploring the role of TXA in Cranial Pathology | | | | | |
| --- | --- | --- | --- | --- | --- |
| Reference | Population | Design/intervention | Comparators | Outcome | Notes |
| Traumatic Brain Injury | | | | | |
| Brito et al., 2023 ^1^ | *Prehospital Tranexamic Acid Use for Traumatic Brain Injury (TXA) Trial. TBI >15 yo, blunt or penetrating, Glasgow Coma Scale [GCS]score ≤ 12, SBP ≥ 90 within 2 hours of injury*  *N=649; 20 Centers; USA and Canada* | *Quadruple Blind RCT: received TXA (either a 2-g bolus or a 1-g bolus plus 1-g 8-hour infusion, referred to below as 2 g and 1 g group, respectively)* | *Twenty-eight-day and 6-month mortalities, 6-month Glasgow Outcome Scale–Extended, and disability rating scale scores* | *Late administration was associated with higher rates of deep venous thrombosis (0.8 vs. 3.4%,*p*= 0.02), cerebral vasospasm (0% vs. 2%,*p*= 0.01), as well as prolonged EMS transport and need for a prehospital airway (*p*< 0.01).* |  |
| Roberts et al., 2021 ^2^ | *CRASH-3. Adults TBI within 3 hours of injury; GCS ≤ 12 or any intracranial bleeding on CT, and no major extracranial bleeding*  *N=9127; 175 centres; 29 countries* | *RCT; TXA (loading dose 1 g over 10 minutes then infusion of 1 g over 8 hours versus placebo* | *Head injury death in hospital within 28 days of injury in patients* | *Early tranexamic acid treatment reduced head injury deaths. Treatment was cost-effective for patients with mild or moderate traumatic brain injury, or those with both pupils reactive.* | *Early treatment was more effective in mild and moderate head injury but there was no obvious impact of time to treatment in cases of severe head injury.*  *The risk of disability, vascular occlusive events and seizures was similar in both groups.* |
| CRASH-3 Intracranial Bleeding Mechanistic Study Collaborators, 2021 ^3^ | *CRASH-3. Patients with TBI with a GCS of 12 or less or intracranial bleeding on a CT scan prior to randomisation. Patients had a median age of 45 years (IQR 29–63), median systolic blood pressure of 136 mmHg (IQR 120–155), and median GCS of 7 (IQR 3–10) (80% male, 20% female)*  *N=1767; Multi-centre; International* | *Eligible patients were randomly allocated to receive tranexamic acid or matching placebo (0.9% sodium chloride) by intravenous infusion.* | *Outcome data were collected 28 days after randomisation, at discharge from the randomising hospital, or at death (whichever was first) using intention treat principle.*  *Haematoma volume, radiological signs of mass effect* | *Early treatment of patients with a mild to moderate GCS reduces head injury death, but there is no evidence for benefit in patients with a severe GCS. TXA tranexamic acid reduces head injury deaths by reducing intracranial bleeding. Patients with a mild to moderate GCS may be more likely to benefit from tranexamic acid because they have less intracranial bleeding at baseline.* |  |
| Safari et al., 2021 ^4^ | *Patients with TBI and intracerebral heamorrhage (16-65 y), who did not need surgical intervention based on a CT scan excluding GCS = 3, with macroscopic heamaturia, history of anticoagulant medication, active vascular thrombosis or a history of it, hypercoagulative state, congenital or acquired hypercoagulopathy, history of vascular disease, valvular heart disease, renal failure, diabetes mellitus, being pregnant or breastfeeding, having a history of an allergic reaction to TXA, history of seizure or brain stroke,*  *N= 95; Single Centre; Iran* | *RCT; intravenous TXA was administered as 1g of bolus and 1g every 6 hours for 48 hours, and in the control group, the placebo was administered in the same way.* | *After 6, 24, and 48 hours all the cases underwent a brain CT scan. Scans were examined for the size and diameter of hematoma and the midline shift. The information regarding the level of consciousness, hematoma volume, and diameter on CT scan were recorded on arrival and 48 hours later.* | *When evaluating hematoma growth rates between the first and last CT scans, it was noted that the mean volume change in the intervention group was significantly lower than in the control group; hematoma growth was 1.17 ± 2.53 ml in the intervention group and 6.85 ± 11.77 ml in the control group. (p=0.002).* |  |
| Anderson et al., 2020 ^5^ | *Prehospital Tranexamic Acid Use for Traumatic Brain Injury (TXA) Trial. TBI >15 yo, blunt or penetrating, Glasgow Coma Scale [GCS] score ≤ 12, SBP ≥ 90 within 2 hours of injury*  *N=287; 20 centers; USA and Canada* | *Quadruple Blind RCT: received TXA (either a 2-g bolus or a 1-g bolus plus 1-g 8-hour infusion, referred to below as 2 g and 1 g group, respectively)* | *Plasma levels of angiopoietin-1, angiopoietin-2, syndecan-1, thrombomodulin, thrombospondin-2, intercellular adhesion molecule 1, vascular adhesion molecule 1.*  *Comparison groups were TXA versus placebo and less than 45 minutes versus 45 minutes or more from time of injury to treatment administration* | *Placebo (n = 129); TXA (n = 158). Levels of syndecan-1 were lower in the TXA group (P = .050 Patients who received TXA less than 45 minutes postinjury had significantly lower levels of angiopoietin-2 (P = .05). No differences were observed in remaining markers.* | *TXA may inhibit early upregulation of syndecan-1 and angiopoietin-2 in patients with MS-TBI, suggesting attenuation of protease-mediated vascular glycocalyx breakdown.* |
| Dixon et al., 2020 ^6^ | *Prehospital Tranexamic Acid Use for Traumatic Brain Injury (TXA) Trial. TBI >15 yo, blunt or penetrating, Glasgow Coma Scale [GCS]score ≤ 12, SBP ≥ 90*  *N=700; 20 centers; USA and Canada* | *Post-hoc analysis RCT: received TXA (either a 2-g bolus or a 1-g bolus plus 1-g 8-hour infusion, referred to below as 2 g and 1 g group, respectively)* | *TEG was performed and coagulation measures including prothrombin time (PT), activated partial thromboplastin time (aPTT), international ratio (INR), fibrinogen, D-dimer, plasmin anti-plasmin (PAP), thrombin anti-thrombin (TAT), tissue plasminogen activator (tPA), and plasminogen activator inhibitor-1 (PAI-1) were quantified at admission and six hours later.* | *There were no statistically significant differences in TEG values, LY30, PT, aPTT, INR, fibrinogen, TAT, tPA, and PAI-1 were demonstrated across treatment groups.*  *Concentrations of D-dimer in TXA treatment groups were less than placebo at six hours (*p*<0.001). Concentrations of PAP were less in TXA treatment groups than placebo on admission (*p*<0.001)* |  |
| Harmer at al., 2020 ^7^ | *Adults ≥ 15y with suspected TBI with a normal CT head. Also GCS score ≤ 12 prior to randomization and administration of sedatives or paralytics, systolic blood pressure ≥ 90 mmHg before randomization, IV access before hospital arrival, estimated age excluding GCS 3 with nonreactive pupils, estimated time of injury greater than 2 hours or unknown time of injury, CPR prior to randomization, burns > 20% of total body surface area, police custody, suspected or known pregnancy, administration of TXA or pro-coagulant prior to randomization, and subjects who chose to “opt-out” based on local regulatory board guidelines*  *N = 395; Multi-centre; Canada and USA* | *Post-Hoc analysis of RCT: TXA was given as 2-gram bolus, 1-gram bolus plus 1-gram infusion over 8 hours, and a placebo bolus with placebo infusion.* | *Fifteen adverse events (28-day incidence) were studied: MI, DVT, seizure, pulmonary embolism, ARDS, cardiac failure, liver failure, renal failure, CVA, cardiac arrest, cerebral vasospasm, “any thromboembolism”, hypernatremia, AKI, and infection. unfavorable outcomes analyzed include mortality at 28 days & 6 months, GOSE ≤ 4 at discharge & 6 months, ICU-free days, ventilator-free days, hospital-free days, and combined unfavorable outcomes.* | *Administration of either a 2-gram TXA bolus or a 1-gram TXA bolus plus 1-gram TXA 8-hour infusion in suspected-TBIs without ICH is not associated with increased adverse events or unfavorable outcomes.* | *Post-hoc analysis* |
| Rowell et al., 2020 ^8^ | *Out-of-hospital patients with TBI aged 15 years or older with GCS <13 and systolic blood pressure of 90 mm Hg or higher. Mean age, 42 years; 255 [74%] male participants; mean Glasgow Coma Scale score, 8.*  *N=819; Multi-centre; USA and Canada* | *Double blinded RTC: Three interventions with treatment initiated within 2 hours of TBI: (1) out-of-hospital tranexamic acid (1 g) bolus and in-hospital tranexamic acid (1 g) 8-hour infusion (bolus maintenance group; n = 312) (2) out-of-hospital tranexamic acid (2 g) bolus and in-hospital placebo 8-hour infusion (bolus only group; n = 345), (3) out-of-hospital placebo bolus and in-hospital placebo 8-hour infusion (placebo group; n = 309).* | *The primary outcome was Glasgow Outcome Scale-Extended score >4 [moderate disability or good recovery] at 6 months in the combined tranexamic acid group vs the placebo group.*  *There were Secondary end points including: 28-day mortality, 6-month Disability Rating Scale score (range, 0 [no disability] to 30 [death]), progression of intracranial hemorrhage, incidence of seizures, and incidence of thromboembolic events.* | *GOS-E>4 in 65% of patients in the tranexamic acid groups vs 62% in the placebo group (difference, 3.5%; [90% 1-sided confidence limit for benefit, −0.9%]; P = .16;* | *There was no statistically significant difference in 28-day mortality, 6-month Disability Rating Scale score or progression of intracranial heamorrhage between the tranexamic acid groups vs the placebo* |
| Brenner et al., 2020 ^9^ | *CRASH-3; <3 h of injury; GCS ≤ 12 or any intracranial bleeding on CT scan and no significant extra-cranial bleeding were eligible. Excluding GCS =3 or bilateral unreactive pupils*  *N=7637* | *randomly assigned (1:1) patients to receive tranexamic acid (loading dose 1 g over 10 min then infusion of 1 g over 8 h) or matching placebo* | *all-cause mortality within 24 h of injury and within 28 days* | *The risk of early death was reduced with TXA (112 (2.9%) TXA group vs 147 (3.9%) placebo group; risk ratio [RR] RR 0.74, 95% CI 0.58–0.94). The risk of death beyond 24 h of injury was only significant once participants from CRASH 2 and 3 were pooled (RR 0.88, 95% CI 0.82–0.94).* | *There was no heterogeneity according to severity and country income* |
| Ebrahimi et al., 2019 ^10^ | *Patients with subdural and epidural hemorrhage*  *N=80; single centre; Iran* | *Double blind RCT: two groups of TXA and 0.9% normal saline as placebo* | *The rate of intracranial hemorrhage after surgery was assessed by CT-scan and amount of hemoglobin (Hb) was measured immediately before surgery and after 6 hours of surgery.* | *Mean of intraoperative bleeding during surgery in patients receiving TXA and placebo in both SDH (Subdural hematoma) and EDH (Epidural Hemorrhage) groups (P= 0.012). The Hb drop amount had no significant difference with placebo (P< 0.0001).* | *No complications were observed in any of the intervention and control groups during the study as well.* |
| Chakroun-Walha et al., 2019 ^11^ | *>18 y with intracranial bleeding on CT-scan, <24 h after trauma, excluding those with significant extracranial injury*  *N=180; single centre; Tunisia* | *Open label randomised trial; Intravenous TXA was administered as soon as possible after randomisation, with a first dose of 1 g in 100 mL of normal saline in 10 min and then with a maintenance dose of 1 g per 500 mL of normal saline for 8 h versus no TXA* | *Neurosurgical intervention or transfusion and the mortality rate up to 28 days after trauma.*  *The secondary endpoints: incidence of VTE and Glasgow Outcome Scale (GOS) at day-28 post trauma.* | *The needs of transfusion or neurosurgery, the mortality rate, the in-hospital length of stay and the dependency at 28-post-traumatic day were similar in the two groups of patients. However, pulmonary embolism was statistically more frequent in ‘TXA’ group (11.5% versus 2.4%, p = 0.02).* |  |
| Fakarian et al., 2018 ^12^ | *TBI with any kind of blood on their computed tomography scan who arrived at the hospital within 8 hours oftrauma, aged 15 and older with nonpenetrating injury*  *N=149; Single centre; Iran* | *Double blind RCT: intravenous TXA 1 g in 10minutes and then with a maintenance dose of 1 gram per 1000 mL of normal saline for 8 hours.* | *The primary outcome was growth of the hemorrhagic lesion on CT at 24 hours* | *The incidence of hemorrhagic lesion growth was 20.5% in the TXA group and 22.7% in the placebogroup. The difference was not significant. The mean hemorrhagic lesion growth was 9.4 (SD 15.3) in the TXA group and 10.2 (SD 10.1).No side effect was observed with the administration of TXA.* | *The limitations of this study were the small sample size and elimination of patients with coagulopathy, which should be considered in subsequent studies. Its strengths include double-blind design, randomized block allocation, precise tomographic data, and 3-month patient follow-up for outcome assessment. None of the patients were excluded from the follow-up period* |
| Valle et al., 2014 ^13^ | *Consecutive trauma patients (n = 1,217) who required emergency surgery (OR) and/or transfusions were reviewed. At surgeon discretion,*  *N=1,217; Single centre; USA* | *Retrospective propensity score matching study comparing TXA with control: TXA was administered at a mean of 97 minutes (1-g bolus then 1-g over 8 hours) to patients deemed high risk. With the use of propensity scores based on age, sex, traumatic brain injury (TBI), mechanism of injury, systolic blood pressure, transfusion requirements, and Injury Severity Score (ISS), these patients were matched to non-TXA patients.* | *Blood product requirements and mortality* | *No differences were found in mortality or fluid requirements between those TBI patients who did and did not receive TXA. However, the sample size of TBI patients in our study is much smaller, and we are likely not sufficiently powered to detect these differences.*  *For the highest injury acuity patients, TXA was associated with increased, rather than reduced, mortality, no matter what time it was administered.* | *This lack of benefit can probably be attributed to the rapid availability of fluids and emergency OR at this trauma center.* |
| Perel et al., 2012 ^14^ | *CRASH 2. Adult trauma patients with significant haemorrhage (systolic blood pressure <90mmHg or heart rate >110 beats per min, or both), or who were considered to be at risk of significant haemorrhage, and who were within 8h of injury, with GCS ≤14 and CT compatible excluding pregnant women and patients for whom a second brain CT scan was not possible*  *N=270; 10 centres; India and Columbia* | *A double-blind, RCT; 1 g of TXA infused over 10 minutes, followed by an intravenous infusion of 1 g over 8 hours or matching placebo (sodium chloride 0.9%)* | *The primary outcome was the occurrence of total haemorrhage growth*  *Secondary outcomes haemorrhage growth defined as an increase by 25%, new/worsening intracranial haemorrhage/mass effect; and new focal ischaemic lesions* | *TXA 133 and Placebo 137. The adjusted analysis showed a greater reduction in total haemorrhage growth in the TXA group than in the placebo group [–3.8 ml, 95% credibility interval (CI) –11·5 ml to 3.9 ml]. In patients who had neurosurgical evacuation before the second CT scan, the extent of this reduction was even larger (–15 ml, 95% CrI –45.7 ml to 15.5 ml). No difference in neurosurgical intervention Reduced risk of poor outcome with TXA (OR 0·59, 95% CrI 0.37 to 0.96).* | *A beneficial effect of TXA was highly probable (range 89% to 94%) for all of the binary CT scan outcomes. The sensitivity analysis for significant haemorrhage growth gave an adjusted odds ratio (OR) of 0·53 (95% CrI 0.41 to 0.68) with a very high probability (99%) of a clinical significant beneficial effect. The sensitivity analysis for new focal cerebral ischaemic lesions was nonsignificant. The probability of a clinically significant harmful effect was 35%.* |
| CRASH 2 Collaborators, 2011 ^15^ | *270 adult trauma patients with, or at risk of, significant extracranial bleeding within 8 hours of injury, who also had traumatic brain injury.*  *N=249; Multi-centre; India and Columbia* | *RCT; Patients randomly allocated to tranexamic acid (loading dose 1 g over 10 minutes, then infusion of 1 g over 8 hours) or matching placebo.* | *Intracranial haemorrhage growth (measured by computed tomography) between hospital admission and then 24–48 hours later, with adjustment for Glasgow coma score, age, time from injury to the scans, and initial haemorrhage volume.* | *No significant difference in haematoma growth, number of new ischaemic lesions, or mortality* |  |
| Spontaneous intracerebral haemorrhage | | | | | |
| Polymeris et al., 2023 ^16^ | *Patients with NOAC-ICH within 12 hours of symptom onset and 48 hours of last NOAC*  *Median age of participants 82 years*  *N=63; 6 centres; Switzerland* | *Double-blind, RCT: intravenous TXA (1 g over 10 minutes followed by 1 g over 8 hours) or matching placebo in addition to standard medical care* | *Primary outcome was hematoma expansion, defined as ≥33% relative or ≥6 mL absolute volume increase at 24 hours and analyzed using logistic regression adjusted for baseline hematoma volume on an intention-to-treat basis.* | *The primary outcome did not differ between TXA (n=32) and placebo (n=31).*  *Between the TXA and placebo arms, the proportion of participants who died or had major thromboembolic complications within 90 days did not differ.* | *There was a signal for interaction with onset-to-treatment time (P=0.024), favoring TXA when administered within 6 hours of symptom onset.*  *All thromboembolic events occurred at least 2 weeks after study treatment, exclusively in participants not restarted on oral anticoagulation.* |
| Law et al., 2021 ^17^ | *TICH-2. Adults with acute spontaneous ICH, presenting within 8 hours of ictus.*  *N=2325; Multi-centre; International* | *RCT; TXA (intravenous 1 g bolus, 1 g infusion/8 h) or placebo* | *Neurological deterioration (increase National Institutes of Health Stroke Scale of ≥ 4 points or a decline in GCS of ≥ 2 from baseline assessed at day 2 (early) and day 7 (late).*  *Hematoma expansion defined as an increase in intraparenchymal hematoma volume on follow-up scan (at 24 h) of > 33% or > 6 mL from baseline volume.* | *TXA reduced neurological deterioration within 7 days (aOR 0.79, 95%CI 0.64–0.97; p = 0.026) and early (aOR 0.79, 95%CI 0.63–0.99; p = 0.041) but not late neurological deterioration*  *TXA reduced the risk of hematoma expansion (aOR 0.76, 0.62–0.93; p = 0.008) and hematoma progression (aOR 0.71, 0.59–0.86; p < 0.001) but not oedema growth at 24 h.* |  |
| Oversen et al., 2021 ^18^ | *TICH2. Spot-sign positive spontaneous ICH in adults presenting to hospital within 8 hours participating in the TICH-2 trial.*  *N=215; Multi-centre; international* | *As per TICH 2: 2 g of tranexamic acid versus matching placebo* | *Primary outcome was absolute day-2 intraparenchymal hematoma volume and Day-2 intraparenchymal and intraventricular hematoma volume.* | *Presence of a spot sign did not modify the treatment effect of tranexamic acid versus placebo.* | *There was low statistical power as well as treatment delay in participants receiving CT angiography* |
| Mousavinejad et al., 2020 ^19^ | *Adults with contusional brain injury presenting within 8 hours of onset*  *N=40; single centre; Iran* | *Double blind RCT: receiving TXA and 0.9% normal saline as a placebo* | *Intracranial hemorrhage volume after surgery using brain CT-scan; hemoglobin (Hb) volume before, immediately after, and six hours after surgery; and the severity of TBI based on Glasgow Coma Score (GCS).* | *Mean hemorrhage during surgery in patients receiving TXA was lower than the placebo group, no significant difference was observed between two groups (P=0.83). Mean Hb volume reduction immediately during surgery and six hours after surgery was also lower in TXA group but had no significant difference.* |  |
| Meretoja et al., 2020 ^20^ | *>18 yo with a spot sign, > 4·5 h of symptom onset, excluding massive haematomas and moribund patients.*  *N=100; 12 centres in Australia, Finland, and Taiwan* | *RCT; TXA 1 g in 100 mL 0·9% NaCl over 10 min followed by 1 g over 8 h or placebo started within 4·5 h of symptom onset.* | *The primary outcome was intracerebral haemorrhage growth (>33% relative or >6 mL absolute) at 24 h. The primary and safety analyses were done in the intention-to-treat population.* | *No evidence that tranexamic acid prevents intracerebral haemorrhage growth, although the treatment was safe with no increase in thromboembolic complications.* |  |
| Sprigg et al., 2019 ^21^ | *TICH-2. Adult patients (aged ≥ 18 years) with ICH within 8 hours of onset excluding secondary ICH (anticoagulation, thrombolysis, trauma, known underlying structural abnormality) prestroke dependence (modified Rankin Scale [mRS] score > 4), life expectancy < 3 months or GCS < 5.*  *N=2307; Multi-centre; Denmark, Georgia, Hungary, Ireland, Italy, Malaysia, Poland, Spain, Sweden, Switzerland, Turkey and UK (Majority)* | *Double Blind RCT; 1 g of an intravenous tranexamic acid bolus followed by an 8-hour 1-g infusion or matching placebo (i.e. 0.9% saline).* | *The primary outcome was functional status (death or dependency) at day 90, measured by mRS score, using ordinal logistic regression, with adjustment for stratification and minimisation criteria.* | *TXA, n = 1152; placebo, n = 1155. There was no statistically significant difference in functional status at day 90 [adjusted odds ratio (aOR) 0.88, 95% confidence interval (CI) 0.76 to 1.03; p = 0.11].*  *There were fewer deaths by day 7 in the tranexamic acid group but no difference in case fatality at 90 days* | *Fewer patients experienced serious adverse events (SAEs) after treatment with tranexamic acid than with placebo by days 2 and 7 (There was no increase in thromboembolic events or seizures.* |
| Sprigg et al., 2014 ^22^ | *Single centre (UK); Adults, excluding secondary ICH (anticoagulation, known vascular malformations) and those with previous vascular occlusive events or VTE*  *N=24, Single centre (UK)* | *RCT; TXA (intravenous 1 g bolus, 1 g infusion/8 h) or placebo within <24 hours of ictus* | *Trial feasibility (primary); Secondary thromboembolic events; functional outcome; NIHSS; haematoma growth* | *16 (TXA) versus 8 participants; trial was feasible; no differences in secondary outcomes* |  |
| Subarachnoid Haemorrhage | | | | | |
| Post et al., 2021 ^23^ | *ULTRA. Adult patients with spontaneous CT-proven subarachnoid haemorrhage excluding peri-mesencephalic bleeding pattern on CT in combination with a Glasgow Coma Scale score of 13–15, and without loss of consciousness directly after ictus or focal neurological deficit on admission; traumatic SAH haemorrhage pattern on CT; ongoing treatment for deep vein thrombosis or pulmonary embolism; a history of a hypercoagulability disorder; pregnancy; severe renal failure or imminent death within 24 h.*  *N=995; Multi-centre; Netherlands* | *RCT: Open-label trial with masked outcome assessment randomly. Treatment with tranexamic acid in addition to care as usual (TXA) or care as usual only (control group). Tranexamic acid was started immediately after diagnosis in the presenting hospital (1 g bolus, followed by continuous infusion of 1 g every 8 h, terminated immediately before aneurysm treatment, or 24 h after start of the medication, whichever came first). TXA was discontinued in non-aneurysmal SAH after diagnostic work-up* | *The primary endpoint was clinical outcome at 6 months, assessed by the modified Rankin Scale, dichotomised into a good (0-3) or poor (4-6) clinical outcome* | *480 patients were randomly assigned to tranexamic acid and 475 patients to the control group.*  *Good clinical outcome was observed in 287 (60%) of 475 patients in the tranexamic acid group, and 300 (64%) of 470 patients in the control group (treatment centre adjusted odds ratio 0·86, 95% CI 0·66-1·12).*  *Rebleeding after randomisation and before aneurysm treatment occurred in 49 (10%) patients in the tranexamic acid and in 66 (14%) patients in the control group (odds ratio 0·71, 95% CI 0·48-1·04).*  *Other serious adverse events were comparable between groups.* |  |
| Post et al., 2019 ^24^ | *SAH confirmed by CT-scan on admission, or by in CSF, with a causative aneurysm angiography who present within 24 hours of symptom onset*  *N=628; 2 centres; Netherlands* | *Retrospective analysis of a prospectively collected database: patients who received TXA versus those who did not* | *Demographic and clinical characteristics, in-hospital complications and clinical outcome were compared between the those who received TXA and those who did not.* | *N=199. In-hospital mortality, was significantly lower in the TXA group than the standard care group (adjusted OR [aOR] 0.42, 95% CI: 0.20–0.85). Poor outcome (mRS 4–6) assessed after six months was not different between treatment groups (aOR 1.05, 95% CI: 0.64–1.74)* |  |
| Hillman et al., 2002 ^25^ | *>15y, CT-verified SAH within 48 hours prior to the first hospital admission excluding pregnancy, history of thromboembolic disease*  *N=254; 3 centres; Sweden* | *Open, randomized trial. A 1-g dose of tranexamic acid was given intravenously as soon as diagnosis of SAH had been verified in the local hospitals (before the patients were transported), followed by doses of 1 g every 6 hours until the aneurysm was occluded; this treatment did not exceed 72 hours* | *Outcome was assessed at 6 months post-SAH by using the Glasgow Outcome Scale (GOS). Vasospasm and delayed ischemic neurological deficits were classified according to clinical findings as well as by transcranial Doppler (TCD) studies. All events classified as rebleeding were verified on CT scans or during surgery.* | *A reduction in the rebleeding rate from 10.8 to 2.4% and an 80% reduction in the mortality rate from early rebleeding with TXA treatment can therefore be inferred.*  *Favorable outcome according to the GOS increased from 70.5 to 74.8%.*  *No increased risk of either ischemic clinical manifestations or vasospasm due to TXA* | *Age, sex, Hunt and Hess and Fisher grade distributions, as well as aneurysm locations, were congruent between the groups*  *A cluster of 27 very early rebleeds still occurred in the control group within hours of randomization into the study, and 13 of these patients died.* |
| Tsementzis et al., 1992 ^26^ | *SAH confirmed by CT scanning and by lumbar puncture*  *N=28; Single centre; UK* | *Case-Control study Nineteen randomly selected patients were studied, 9 receiving tranexamic acid (9 g a day) and the remaining 10 placebo* | *The intravenous Xe133 technique was used for serial determinations of hemispheral cerebral blood flow.* | *CBF was stable during the first week after SAH then fell progressively reaching its nadir by the end of the second week. There was a rebound in CBF in both cerebral hemispheres. CBF was greatest in the contralateral (to side of ruptured aneurysm) brain hemisphere. The difference between the ipsi-and contralateral hemispheres was most pronounced in patients receiving TXA* |  |
| Tsementzis et al., (1990) ^27^ | *SAH confirmed with LP (xanthochromic CSF) or CT brain scan.*  *N=100; Single centre: UK* | *Doubled blind RCT; 9g a day in six doses, until successful surgery or four weeks from ictus, whichever was the sooner.*  *Tranexamic acid was given 4 hours in half hour infusions of 1.5g in 50ml of saline for one week and then 3 tablets (0.5 g each) every 4 hours for the remaining 3 weeks. This group received the placebo treatment in an otherwise identical manner.* | *Rebleed defined as sudden deterioration with confirmation on CT or LP or autopsy; Glasgow Outcome Score at discharge one, three and six monthly intervals after discharge; Vasospasm on angiography; Delayed Cerebral Ischaemia defined as new or worsening consciousness impairment, focal neurological deficits in the absence of any evidence of other possible causes including recurrent haemorrhage, hypoxia or electrolyte disturbance; Side-effects of TXA: nausea, vomiting, diarrhoea, behavioral changes, visual disturbance, DVT and PE* | *Rates of rebleed and death from rebleed were similar between TXA nd placebo at 12% and 5-7% respectively.*  *Incidence of cerebral infarction overall was greater in the TXA group (27%) than in the control group (11%).*  *Disability due to vasospasm (p less than 0.04); the reverse was true for the placebo patient (p less than 0.05).* | *Post-operative cerebral ischaemia was significantly more frequent in the TXA group. In a fifth of the patients in whom cerebral blood flow was estimated there was a significant reduction of cerebral blood flow (CBF) on the side of the ruptured aneurysm in the TXA treated group.* |
| Tsementzis et al., 1990 ^28^ | *SAH confirmed with LP (xanthochromic CSF) or CT brain scan.*  *N=74; single centre; UK* | *RCT: 9 grams per day of TXA by intravenous infusion for 21 days or until operation. The control patients received a placebo infusion.* | *Blood and CSF samples were taken before treatment, and then weeks 1 week ad 2 weeks.*  *Plasminogen activity* | *Complications such as rebleeding, hydrocephalus or cerebral thrombosis could not be predicted from analysis of fibrinolytic activity. Tranexamic acid treatment resulted in a reduction in cere- brospinal fluid and blood plasminogen activity.* |  |
| Jordan, 1985 ^29^ | *Summary of 2 controlled trials of TXA in patients with subarachnoid haemorrhage. Patients had aneurysms clipped an average of 16 days following haemorrhage.*    *N=105; 2 centres; UK and Netherlands* | *RCT; TXA versus control, unblinded.*  *TXA administered orally or intravenously* | *Death from cerebral ischaemia or rebleed* | *25% mortality versus 19% in TXA versus control.*  *With more deaths from cerebral ischaemia at median follow-up of 25 months* | *More arachnoid adhesions in patients on TXA*  *No statistical analysis. Note peak TXA levels were achieved after 48 hours of administrations in CSF* |
| Vermeulen et al., 1984 ^30^ | *Patients with SAH confirmed on LP, CT or angiography, excluding those with VTE, pregnant, coagulopathy presenting >72 hours post haemorrhage*  *N=479; 4 centres: Netherlands and UK*  *.* | *Double-blind, RCT; placebo or TXA started within 72 hours of haemorrhage (4-6 g TXA intravenously or orally (intravenously at least for the first 2 weeks). Treatment was discontinued at surgery or* | *Three month Glasgow Outcome Scale post discharge*  *Incidence of rebleed (definite or possible) and infarction (definite or possible)* | *At 3 months, no difference in outcomes or mortality between TXA and placebo. Rebleeding was reduced from 24 per cent in the control group to 9 per cent in the tranexamic acid-treated group (chi-square = 18.07, P<0.001). But, there was an increase in the incidence of ischemic complications (15 per cent in the control group and 24 per cent in the tranexamic acid group; chi-square = 8.07, P<0.01).* | *There was no significant difference between the groups in aneurysmal SAH* |
| Muizelaar et al., 1988 ^31^ | *Patients with SAH aged 66 or older who are mostly conservatively managed (95%)*  *N=66; 4 centres; UK and Netherlands* | *Double blind RCT: TXA within 72 hours from the first SAH; 6 x 1 IV in the first week and 4 x 1 g IV in the second, followed in the next two weeks by 4 x 1 g IV The dosage was different after the the initial first week in the Netherlands (4g/24 hour IV) and UK (6g/24h by mouth)* | *Mortality, rebleed, cerebral infarction, hydrocephalus, GOS, GOS* | *Death from rebleed: 21% in the drug group and 62% in the placebo group*  *Death by cerebral infarction four times more common in TXA group*  *High frequency of hydrocephalus in TXA group* |  |
| Mendelow et al., 1982 ^32^ | *Diagnosis of subarachnoid haemorrhage had been proven by lumbar puncture and/or CAT scan who were administered TXA*  *N= 53; 1 Centre; Scotland* | *RCT; Aspirin or placebo was given orally twice daily and in patients unable to swallow administration was by rectal retention enema.* | *Platelet aggregation*  *Neurological deficit death* | *Low dose aspirin does not affect the outcome in patients with subarachnoid haemorrhage being treated with the anti-fibrinolytic agent tranexamic acid* |  |
| Chowdary and Sayed, 1981 ^33^ | *Patients with SAH 19% No aneurysm and 5% AVM. Two thirds female. 80-90% Hunt&Hess I or II*  *N=151; Single Centre; Ireland* | *Pseudo-randomised trial: Epsilon aminocaproic acid 36g/day versus TXA 6g/day. Oral or intravenous until surgery. 70-80% started within 7 days of ictus* | *Rebleed, death from rebleed, delayed ischaemic deficit, mortality* | *91 patients epsilon aminocaproic acid; 61 TXA. No difference between outcomes* |  |
| Fodstad and Nilsson, 1981 ^34^ | *Patients with ruptured cerebral aneurysms, N=41; Single centre; Sweden* | *RCT: The drug was administered by slow intravenous injection in a dosage of 1 g every four hours for the first week and 1 g every six hours during the following four weeks versus bedrest and sedation* | *Fibrinolysis in blood and in CSF*  *Change in blood coagulation factors* | *TXA inhibited local fibrinolysis in CSF. TXA reduced the concentration of plasminogen in plasma and a2-macroglobulin in CSF, but had no effect on other coagulation factors. An increase in local fibrinolysis in CSF occurs after rebleeding and in patients with symptoms of delayed cerebral ischaemia* |  |
| Kaste and Ramsay, 1979 ^35^ | *Diagnosis of SAH (acute headache, neck rigidity) confirmed on LP; <61 years; excluding unconscious, recent MI, renal failure, DIC and pregnancy.*  *N=64; single centre, Finland* | *RCT; double blinded; Daily doses of 6 g TXA starting within 72 hours of symptom onset after diagnosis* | *Rebleed prior to aneurysm clipping, or 21 days whichever longest. Mortality. Thrombo-embolic events, mortality and morbidity* | *TXA = 32 versus P= 32; 25 and 24 aneurysmal respectively; no difference in re-bleeds, mortality between the tranexamic and placebo-treated groups. No thromboembolic complications were noted in either group.* |  |
| Chandra, 1978 ^36^ | *Subarachnoid haemorrhage confirmed on CSF with an angiographically demonstrated aneurysm*  *N=39; Single centre; Indonesia* | *Twenty patients received tranexamic acid, 6 gm daily for 14 to 2 1 days, while 19 patients received conventional therapy of bedrest and dexamethasone when cerebral edema developed, plus isotonic saline.* | *Rebleeding and mortality* | *Rebleeding and mortality were reduced by one-fourth and ow-fifth, respectively (p < 0.01).*  *No side-effects were observed* |  |
| Maurice-Williams, 1978 ^37^ | *Under 65 and without intercurrent disease, (b) relatively little disturbed by the first bleed (Botterell grades l-3) and admitted within 96 hours of the first haemorrhage excluding non-aneurysmal SAH*  *N=50; Single centre; UK* | *Open label, randomised controlled trial; 6 g/day TXA for 42 days or operation or bed rest and sedation* | *Death and number of rebleeds* | *12% (TXA) versus 40% (control) death by rebleed (p-value 0.025)*  *6 (TXA) vs 14 (control) rebleeds*  *3 (TXA) vs 11 (control) deaths* |  |
| Van Rossum et al., 1977 ^38^ | *A diagnosis of SAH based on haemorrhagic spinal fluid not caused by lumbar puncture and severe headache of acute onset, accompanied by neck rigidity.*  *N=51; 3 centres; Netherlands* | *Double blind RCT; administration of either intravenous placebo or drug (1g TXA every 6 hours) for ten consectuive days. 37/51 patients within 48 hours.* | *Mortality and neurological condition at 3 months* | *No difference in death or neurological condition at 3 months (drowsy, alert, coma)* |  |
| Meningioma Surgery | | | | | |
| [Hood](https://doi.org/10.1016/j.jocn.2017.02.053)a et al., 2017^39^ | *Patients undergoing elective meningioma surgery excluding coagulopathy, thrombophilia, pre-operative embolization, small tumours (<4 cm) or those with minimal expected blood loss*  *N=60; single centre; India* | *RCT; intra-operative 2 g TXA loading dose before skin incision followed by a maintenance infusion of 0.025 ml/kg/h versus placebo .* | *Intraoperative blood loss and transfusion requirements, estimation using a 5-grade heamostasis scale were recorded during meningioma excision performed by a neurosurgeon with >3 y experience* | *Greater degree of intra-operative blood loss (1.3 times greater compared to TXA). Increased surgical field bleeding and greater volume of post-operative transfusion required in placebo group* |  |
| Craniosynostosis | | | | | |
| Fenger-Eriksen et al., 2020 ^40^ | *Children ASA grade I/II scheduled for craniosynostosis surgery Average age and weight were 1.4 years (range, 0.35-9.1 years), and 10.7 kg (range, 6.1-39 kg).*  *N=30; single centre; Denmark* | *RCT; TXA (bolus dose of 10 mg/kg injected prior to first surgical incision, followed by 8 hours continuous infusion of 3 mg/kg/h) or an equivalent volume of placebo* | *Temporal and placebo vs TXA comparisons of serum chemokines, interleukins, cell surface marker proteins, growth factors, tumour necrosis factor, macrophage stimulation, leukaemia inhibitors, monocyte chemo-attractants* | *TXA administration in a low-dose regimen including bolus followed by 8 hours infusion during craniosynostosis surgery did not change any of 92 inflammatory markers as compared with placebo.* |  |
| [Goobie](https://doi.org/10.1016/j.bja.2020.05.054) et al., 2020 ^41^ | *3 m - 2 y undergoing cranial remodelling surgery for craniosynostosis (fronto-orbital advancement surgery, and posterior and total calvarial remodelling procedures). Excluding those with coagulopathy, or recent acetylsalicylate or NSAID ingestion*  *N=68; two centres USA* | *RCT double-blind non- inferiority; Intra-operative IV TXA (50 mg/kg loading dose versus 10 mg/kg over 15 min followed by a maintenance infusion of 5 mg/kg/h)* | *Intraoperative blood loss*  *blood product transfusion, biological markers of fibrinolysis and inflammation, and TXA plasma concentrations. Complications (seizures or thromboembolic events) and patient-centred outcomes (ICU and hospital length of stay) are also reported.* | *Tranexamic acid 10 mg/kg followed by 5 mg/kg/h is not less effective than a higher dose of 50 mg/kg and 5 mg kg/h in reducing blood loss and transfusion in paediatric craniosynostosis surgery.* | *No quantitative difference in concentrations of markers of fibrinolysis or inflammation between the high-dosage and low-dosage TXA schedule* |
| Fenger-Eriksen et al., 2019 ^42^ | *Children ASA classification ½ mean age 1.4 yr and 10.7 kg excluding any known bleeding disorders, reduced renal function, history with unexplained seizures, and known allergy for TXA.*  *N=30; single centre; Denmark* | *RCT; TXA (bolus dose of 10 ml kg−1 injected before the first surgical incision, followed by 8 h continuous infusion of 3 ml/kg/h) or an equivalent volume of placebo (isotonic saline, B. Braun, Frederiksberg, Denmark)* | *The primary endpoint was postoperative blood loss measured in drainage canisters from the end of surgery and during the following 24 h measured from drainage canisters were placed during skin closure at the end of surgery.*  *Secondary end point: blood loss during surgery, transfusion requirements, haemoglobin and coagulation parameters, volume fluid infusion, duration of surgery. Clot stability and fibrinolysis were investigated by the fibrin clot lysis assay, thromboelastometry, and coagulation factor XIII (FXIII) concentrations.* | *TXA reduced postoperative blood loss by 18 ml/kg  (*P*<0.001). Intraoperative*[*red blood cell*](https://www.sciencedirect.com/topics/medicine-and-dentistry/erythrocyte)*(RBC) and fresh frozen plasma (FFP) transfusions were reduced by 5.8 ml/kg  (*P*=0.01) and from FFP 5.2 ml/kg (*P*=0.03). Postoperative*[*RBC transfusion*](https://www.sciencedirect.com/topics/medicine-and-dentistry/erythrocyte-transfusion)*median was 5 (inter-quartile range [IQR] 0–6) ml/kg in the placebo group and 0 (0–5.7) ml/kg in the TXA group. Resistance to lysis was higher in the treatment group (*P*<0.001).* |  |
| Kim et al., 2018 ^43^ | *Children 4m-5 y undergoing distraction osteogenesis, excluding thrombocytopenia, coagulopathy, epileptics, previous brain surgery, use of aspirin and NSAIDs.*  *N= 48; Single Centre; Korea* | *RCT ; TXA (or placebo) was administered as an intravenous loading dose of 10 mg/kg over 15 min, followed by continuous intravenous infusion at 5 mg/kg/h from the beginning of the surgery until skin closure* | *Estimated blood loss (EBL)*  *Thrombo-elastometry*  *D-dimer*  *VTE*  *Neurological outcome at 3 months*  *Mechanical ventilation,*  *Transfusion requirements* | *TXA reduced EBL, transfusion requirements ventilation and attenuated D-dimer increase.* |  |
| Goobie et al., 2013 ^44^ | *Mean age 23 months excluding those with history of haematological abnormalities, coagulation, hepatic, renal or vascular disorders, and NSAID use within 2 days or aspirin within 14 days*  *N= 23; Single centre; USA* | *Phase I study; TXA administered intra-operatively TXA was administered at an IV dose of 50 mg/kg over 15 min, followed by a 5 mg/kg/h intravenous maintenance infusion for the duration of the surgery* | *Serum TXA levels versus time*  *Pharmacokinetic modelling*  *Estimated blood glucose* | *Modelling suggested that a lower loading dose of 10 mg/kg over 15 min followed by a 5 mg/kg/h maintenance infusion can avoid high initial peaks, and still achieve therapeutic serum concentrations (>16 μg/mL).* |  |
| Goobie et al., 2011^45^ | *Children (age range, 2 months to 6 yr) undergoing craniosynostosis reconstruction surgery excluding those with hematologic abnormalities or coagulation, hepatic, renal, or vascular disorders or ingestion of nonsteroidal antiinflammatory agents or acetylsalicylic acid*  *N=43; Single Centre; USA* | *After induction of anesthesia and before skin incision, patients received a loading dose of 50 mg·kg^−1^ over infusion over 15 min, followed immediately by an infusion of 5 mg/kg/h (0.1 ml/kg/h). Patients in the placebo group received an equivalent dose of 0.9% saline* | *The primary and secondary outcome variables were reduction in perioperative blood loss and reduction in perioperative blood transfusion, respectively*  *Also:*  *Measurement of TXA in plasma*  *Thromboelastography* | *The TXA group had significantly lower perioperative mean blood loss (65 vs. 119 ml/kg, P < 0.001) and lower perioperative mean blood transfusion (33 vs. 56 ml/ kg, P = 0.006) compared to the placebo group. TXA administration also significantly diminished (by two thirds) the perioperative exposure of patients to transfused blood (median, 1 unit vs. 3 units; P < 0.001). TXA plasma concentrations were maintained above the in vitro thresholds reported for inhibition of fibrinolysis (10 μg/ml) and plasmin-induced platelet activation (16 μg/ml) throughout the infusion.* |  |
| Chronic Subdural Haematoma | | | | | |
| Miyakoshi et al., 2023 ^46^ | *Patients >59 years undergoing burr hole drainage of CSDH during 2012-2020 excluding those with dialysis dependent renal failure, middle meningeal artery embolization*  *N=946; Japan; Population based cohort study* | *Retrospective, propensity score–matched population cohort study using a health insurance database; TXA exposure was defined as a TXA prescription ≥14 days from index month, and data on postoperative administration of TXA were collected. The control group was defined as patients who had not been prescribed TXA, who had been prescribed TXA <14 days, or who started a TXA prescription in the months after the index month* | The primary outcome was repeat surgery, and the secondary outcome was death or the onset of thrombosis | *TXA (N=473) matched 1:1 versus control. Repeat surgery was performed in 30 of 465 patients (6.5%) in the TXA group and in 78 of 465 patients (16.8%) in the control group (relative risk, 0.38; 95% CI, 0.26-0.56). No significant difference was observed for death or the onset of thrombosis.* | *Limited to Japanese ethnicity, non-interventional, non- randomised study, unmeasured variables included radiological information, operative technique clinical symptoms., standardised operative approaches* |
| De Paula et al., 2023 ^47^ | *Patients aged over 18 years with unilateral or bilateral chronic subdural hematoma and submitted to surgical drainage by trepanation excluding malignant diseases, history of acute myocardial infarction, stroke, severe peripheral arterial disease, or any other condition that contraindicated the use of tranexamic acid. Patients who needed to be reoperated due to acute rebleeding in the immediate postoperative period (first 12–24 h)*  *N=50; Single centre; Brazil* | *Open label RCT: TXA at a dose of 750 mg/day in 3 daily doses for 3 months or no TXA* | Primary outcome radiological and clinical recurrence of CSDH at 6 months. *Criteria for indication of surgery: symptomatic unilateral or bilateral chronic subdural hematoma or thicker than 10 mm.* | *TXA group had a higher recurrence rate (8.3%), there was no significant difference between both groups.* |  |
| Wan et al., 2020 ^48^ | *Patients admitted for surgical evacuation of their CSDH excluding criteria those with history (or those at risk of) venous thrombo-embolic events, significant comorbidity: other intracranial pathologies, significant renal or liver impairment, pregnancy (for females), concurrent steroid use, at risk for thrombosis, history of hypercoagulability, moribund*  *N=90; 3 centers; Singapore* | *Open-label randomized trial; Patients in the TXA arm were prescribed one capsule of 500 mg TXA twice daily, for a total of 21 days.* | *The primary outcome was the reduction of post-surgery recurrence (symptomatic CSDH recurrence requiring further surgery) within 6 months of surgery*  *Secondary outcomes included volumetric differences at 6, 12 and 24 weeks interval post-operatively.* | *49 patients observation 41 TXA. Patients in the TXA arm demonstrated a greater reduction of their CSDH volume at 6 weeks follow up (36.6%) compared to the observation arm (23.3%, p = 0.6648). There were no reportable serious adverse events recorded in the observation arm, compared to 4 (9.8%) patients in the TXA arm.* | *Significant loss to follow-up*  *Significantly biases toward patients without comorbidity resulting in a pre-selected group of ‘well’ patients.* |
| Yamada and Natori et al., 2020 ^49^ | *A total of 297 patients (354 hematomas) with CSDH underwent initial burr hole surgery at our hospital from April 2014 to March 2018. Of these, 206 patients (250 hematomas) consented to participate in this study.*  *N=206; single centre; Japan* | *Patients were randomly divided into the observation, TXA, and Goreisan groups (traditional Japanese remedy), based on age. In the TXA group, patients took 750 mg of TXA per day in 3 divided doses after each meal. In the Goreisan group, patients took 7.5 g of Goreisan per day in 3 divided doses before each meal. The oral administration intervention was implemented from the day after surgery to 3 months after surgery.* | *The observation period was 3 months after surgery. recurrence rates; reoperation was considered as a recurrence.*  *the volume of residual hematoma and air (subdural pneumocephalus) was compared using head computed tomography (CT) scan at 1 day, 1 week, and 1, 2, and 3 months after surgery.* | *Oral administration of tranexamic acid or Goreisan did not minimize recurrence after chronic subdural hematoma burr hole surgery; however, tranexamic acid can reduce the hematoma volume.* |  |
| Perinatal Periventricular Haemorrhage | | | | | |
| Hensey, Morgan and Cook, 1984 ^50^ | *Infants at risk of periventricular haemorrhage i.e. <1250g at birth infants <1500g who required respiratory support in the first day of life*  *N=100; Single Centre; UK* | *Double- Blind RCT Infants received 0-25 ml/kg of TXA or placebo intravenously six hourly for five days* | *Periventricular haemorrhages developed in 22 infants in the treated group and 20 in the placebo group. The mean time of detection of haemorrhage in both groups was 48 hours.* | *Tranexamic acid does not have a role in the prevention of periventricular haemorrhage in the very low birth weight infant* |  |
| Abbreviations: ASA American Society of Anaesthesiology Grade; CBF Cerebral Blood Flow; CSDH Chronic Subdural Haematoma; CSF Cerebrospinal Fluid; CT Computer Tomography; DCI Delayed Cerebral Ischaemia; DWIHL Diffusion Weighted Imaging Lesions; DVT Deep Vein Thrombosis; EBL Estimated Blood Loss; EVD External Ventricular Drainage; GCS Glasgow Coma Scale; GOS Glasgow Outcome Score; GOS-E Glasgow Outcome Score-Extended; IV Intravenous; LP Lumbar Puncture; mRS modified Rankin Score; NIHSS National Institute of Health Stroke Scale; NSAID Non-Steroidal Anti-Inflammatories; PE Pulmonary Embolus; RBC Red Blood Cell; RCT Randomised Controlled Trial; SBP Systolic Blood Pressure; TEG Thromboelastography; TXA Tranexamic Acis; VTE Venous Thromboembolism | | | | | |

References

1. Brito AM, Schreiber MA, El Haddi J, Meier EN, Rowell SE. The effects of timing of prehospital tranexamic acid on outcomes after traumatic brain injury: Subanalysis of a randomized controlled trial. *Journal of trauma and acute care surgery.* 2023;94(1):86-92.

2. Roberts I, Shakur-Still H, Aeron-Thomas A, et al. Tranexamic acid to reduce head injury death in people with traumatic brain injury: the CRASH-3 international RCT. *Health Technology Assessment (Winchester, England).* 2021;25(26):1.

3. Collaborators C-IBMS. Tranexamic acid in traumatic brain injury: an explanatory study nested within the CRASH-3 trial. *European Journal of Trauma and Emergency Surgery.* 2021;47:261-268.

4. Safari H, Farrahi P, Rasras S, Marandi HJ, Zeinali M. Effect of intravenous tranexamic acid on intracerebral brain hemorrhage in traumatic brain injury. *Turk Neurosurg.* 2021;31(2):223-227.

5. Anderson TN, Hinson HE, Dewey EN, Rick EA, Schreiber MA, Rowell SE. Early tranexamic acid administration after traumatic brain injury is associated with reduced syndecan-1 and angiopoietin-2 in patients with traumatic intracranial hemorrhage. *The Journal of head trauma rehabilitation.* 2020;35(5):317.

6. Dixon AL, McCully BH, Rick EA, et al. TXA administration in the field does not affect admission TEG after traumatic brain injury. *The journal of trauma and acute care surgery.* 2020;89(5):900.

7. Harmer JW, Dewey EN, Meier EN, Rowell SE, Schreiber MA. Tranexamic acid is not inferior to placebo with respect to adverse events in suspected traumatic brain injury patients not in shock with a normal head computed tomography scan: A retrospective study of a randomized trial. *Journal of Trauma and Acute Care Surgery.* 2022;93(1):98-105.

8. Rowell SE, Meier EN, McKnight B, et al. Effect of out-of-hospital tranexamic acid vs placebo on 6-month functional neurologic outcomes in patients with moderate or severe traumatic brain injury. *Jama.* 2020;324(10):961-974.

9. Brenner A, Belli A, Chaudhri R, et al. Understanding the neuroprotective effect of tranexamic acid: an exploratory analysis of the CRASH-3 randomised trial. *Critical Care.* 2020;24(1):1-10.

10. Ebrahimi P, Mozafari J, Ilkhchi RB, Hanafi MG, Mousavinejad M. Intravenous tranexamic acid for subdural and epidural intracranial hemorrhage: randomized, double-blind, placebo-controlled trial. *Reviews on Recent Clinical Trials.* 2019;14(4):286-291.

11. Chakroun-Walha O, Samet A, Jerbi M, et al. Benefits of the tranexamic acid in head trauma with no extracranial bleeding: a prospective follow-up of 180 patients. *European Journal of Trauma and Emergency Surgery.* 2019;45:719-726.

12. Fakharian E, Abedzadeh-Kalahroudi M, Atoof F. Effect of tranexamic acid on prevention of hemorrhagic mass growth in patients with traumatic brain injury. *World Neurosurgery.* 2018;109:e748-e753.

13. Valle EJ, Allen CJ, Van Haren RM, et al. Do all trauma patients benefit from tranexamic acid? *Journal of Trauma and Acute Care Surgery.* 2014;76(6):1373-1378.

14. Perel P, Al-Shahi Salman R, Kawahara T, et al. CRASH-2 (Clinical Randomisation of an Antifibrinolytic in Significant Haemorrhage) intracranial bleeding study: the effect of tranexamic acid in traumatic brain injury--a nested randomised, placebo-controlled trial. *Health technology assessment (Winchester, England).* 2012;16(13):iii-54.

15. Collaborators C-. Effect of tranexamic acid in traumatic brain injury: a nested randomised, placebo controlled trial (CRASH-2 Intracranial Bleeding Study). *Bmj.* 2011;343.

16. Polymeris AA, Karwacki GM, Siepen BM, et al. Tranexamic Acid for Intracerebral Hemorrhage in Patients on Non-Vitamin K Antagonist Oral Anticoagulants (TICH-NOAC): A Multicenter, Randomized, Placebo-Controlled, Phase 2 Trial. *Stroke.* 2023;54(9):2223-2234.

17. Law ZK, Dineen R, England TJ, et al. Predictors and outcomes of neurological deterioration in intracerebral hemorrhage: results from the TICH-2 randomized controlled trial. *Translational stroke research.* 2021;12:275-283.

18. Ovesen C, Jakobsen JC, Gluud C, et al. Prevention of haematoma progression by tranexamic acid in intracerebral haemorrhage patients with and without spot sign on admission scan: a statistical analysis plan of a pre-specified sub-study of the TICH-2 trial. *BMC research notes.* 2018;11(1):1-8.

19. Mousavinejad M, Mozafari J, Ilkhchi RB, Hanafi MG, Ebrahimi P. Intravenous tranexamic acid for brain contusion with intraparenchymal hemorrhage: randomized, double-blind, placebo-controlled trial. *Reviews on Recent Clinical Trials.* 2020;15(1):70-75.

20. Meretoja A, Yassi N, Wu TY, et al. Tranexamic acid in patients with intracerebral haemorrhage (STOP-AUST): a multicentre, randomised, placebo-controlled, phase 2 trial. *The Lancet Neurology.* 2020;19(12):980-987.

21. Sprigg N, Flaherty K, Appleton JP, et al. Tranexamic acid to improve functional status in adults with spontaneous intracerebral haemorrhage: the TICH-2 RCT. *Health technology assessment (Winchester, England).* 2019;23(35):1.

22. Sprigg N, Renton CJ, Dineen RA, Kwong Y, Bath PM. Tranexamic acid for spontaneous intracerebral hemorrhage: a randomized controlled pilot trial (ISRCTN50867461). *Journal of Stroke and Cerebrovascular Diseases.* 2014;23(6):1312-1318.

23. Post R, Germans MR, Tjerkstra MA, et al. Ultra-early tranexamic acid after subarachnoid haemorrhage (ULTRA): a randomised controlled trial. *The Lancet.* 2021;397(10269):112-118.

24. Post R, Germans M, Boogaarts H, et al. Short-term tranexamic acid treatment reduces in-hospital mortality in aneurysmal sub-arachnoid hemorrhage: A multicenter comparison study. *PLoS One.* 2019;14(2):e0211868.

25. Hillman J, Fridriksson S, Nilsson O, Yu Z, Säveland H, Jakobsson K-E. Immediate administration of tranexamic acid and reduced incidence of early rebleeding after aneurysmal subarachnoid hemorrhage: a prospective randomized study. *Journal of neurosurgery.* 2002;97(4):771-778.

26. Tsementzis S, Meyer C, Hitchcock E. 19Cerebral blood flow in patients with a subarachnoid haemorrhage during treatment with tranexamic acid. *Neurochirurgia.* 1992;35(03):74-78.

27. Tsementzis S, Hitchcock E, Meyer C. Benefits and risks of antifibrinolytic therapy in the management of ruptured intracranial aneurysms: a double-blind placebo-controlled study. *Acta neurochirurgica.* 1990;102:1-10.

28. Tsementzis S, Honan W, Nightingale S, Hitchcock E, Meyer C. Fibrinolytic activity after subarachnoid haemorrhage and the effect of tranexamic acid. *Acta neurochirurgica.* 1990;103:116-121.

29. Jordan K. Antifibrinolytic therapy in subarachnoid hemorrhage. *The New England Journal of Medicine.* 1985;312(1):52-53.

30. Vermeulen M, Lindsay K, Murray G, et al. Antifibrinolytic treatment in subarachnoid hemorrhage. *New England Journal of Medicine.* 1984;311(7):432-437.

31. Muizelaar JP, Vermeulen M, van Crevel H, et al. Outcome of aneurysmal subarachnoid hemorrhage in patients 66 years of age and older. *Clinical neurology and neurosurgery.* 1988;90(3):203-207.

32. Mendelow A, Stockdill G, Steers A, Hayes J, Gillingham F. Double-blind trial of aspirin in patients receiving tranexamic acid for subarachnoid haemorrhage. *Acta neurochirurgica.* 1982;62:195-202.

33. U M Chowdhary KS. Comparative clinical trial of epsilon amino-caproic acid and tranexamic acid in the prevention of early recurrence of subarachnoid haemorrhage. *Journal of Neurology, Neurosurgery & Psychiatry.* 1981;44(9):810-813.

34. Fodstad H, Nilsson I. Coagulation and fibrinolysis in blood and cerebrospinal fluid after aneurysmal subarachnoid haemorrhage: Effect of tranexamic acid (AMCA). *Acta Neurochirurgica.* 1981;56:25-38.

35. Kaste M, Ramsay M. Tranexamic acid in subarachnoid hemorrhage. A double-blind study. *Stroke.* 1979;10(5):519-522.

36. Chandra B. Treatment of subarachnoid hemorrhage from ruptured intracranial aneurysm with tranexamic acid: a double‐blind clinical trial. *Annals of Neurology: Official Journal of the American Neurological Association and the Child Neurology Society.* 1978;3(6):502-504.

37. Maurice-Williams R. Prolonged antifibrinolysis: an effective non-surgical treatment for ruptured intracranial aneurysms? *Br Med J.* 1978;1(6118):945-947.

38. Van Rossum J, Wintzen A, Endtz L, Schoen J, De Jonge H. Effect of tranexamic acid on rebleeding after subarachnoid hemorrhage: a double‐blind controlled clinical trial. *Annals of Neurology: Official Journal of the American Neurological Association and the Child Neurology Society.* 1977;2(3):242-245.

39. Hooda B, Chouhan RS, Rath GP, Bithal PK, Suri A, Lamsal R. Effect of tranexamic acid on intraoperative blood loss and transfusion requirements in patients undergoing excision of intracranial meningioma. *Journal of Clinical Neuroscience.* 2017;41:132-138.

40. Fenger‐Eriksen C, Rasmussen M, Juul N, Krog J, Hvas AM. Effect of tranexamic acid on markers of inflammation in children undergoing craniofacial surgery. *Acta Anaesthesiologica Scandinavica.* 2021;65(1):34-39.

41. Goobie SM, Staffa SJ, Meara JG, et al. High-dose versus low-dose tranexamic acid for paediatric craniosynostosis surgery: a double-blind randomised controlled non-inferiority trial. *British journal of anaesthesia.* 2020;125(3):336-345.

42. Fenger-Eriksen C, Lindholm ADA, Nørholt SE, et al. Reduced perioperative blood loss in children undergoing craniosynostosis surgery using prolonged tranexamic acid infusion: a randomised trial. *British Journal of Anaesthesia.* 2019;122(6):760-766.

43. Kim EJ, Kim YO, Shim KW, Ko BW, Lee JW, Koo B-N. Effects of tranexamic acid based on its population pharmacokinetics in pediatric patients undergoing distraction osteogenesis for craniosynostosis: rotational thromboelastometry (ROTEMTM) analysis. *International journal of medical sciences.* 2018;15(8):788.

44. Goobie SM, Meier PM, Sethna NF, et al. Population pharmacokinetics of tranexamic acid in paediatric patients undergoing craniosynostosis surgery. *Clinical pharmacokinetics.* 2013;52:267-276.

45. Goobie SM, Meier PM, Pereira LM, et al. Efficacy of tranexamic acid in pediatric craniosynostosis surgery: a double-blind, placebo-controlled trial. *The Journal of the American Society of Anesthesiologists.* 2011;114(4):862-871.

46. Miyakoshi A, Nakatani E, Kaneda H, et al. Administration of Tranexamic Acid After Burr Hole Craniotomy Reduced Postoperative Recurrence of Chronic Subdural Hematoma in a Japanese Regional Population. *Neurosurgery.* 2023;93(5):1160-1167.

47. de Paula MVCT, Ribeiro BDC, Melo MM, et al. Effect of postoperative tranexamic acid on recurrence rate and complications in chronic subdural hematomas patients: preliminary results of a randomized controlled clinical trial. *Neurosurgical Review.* 2023;46(1):90.

48. Wan KR, Qiu L, Saffari SE, et al. An open label randomized trial to assess the efficacy of tranexamic acid in reducing post-operative recurrence of chronic subdural haemorrhage. *Journal of Clinical Neuroscience.* 2020;82:147-154.

49. Yamada T, Natori Y. Prospective study on the efficacy of orally administered tranexamic acid and goreisan for the prevention of recurrence after chronic subdural hematoma burr hole surgery. *World neurosurgery.* 2020;134:e549-e553.

50. Hensey O, Morgan M, Cooke R. Tranexamic acid in the prevention of periventricular haemorrhage. *Archives of disease in childhood.* 1984;59(8):719-721.
